# Non-invasive closed-loop spinal stimulation restores leg stepping control in humans with paraplegia

**DOI:** 10.1093/brain/awaf230

**Published:** 2025-11-26

**Authors:** Toshiki Tazoe, Syusaku Sasada, Takashi Murayama, Yaoki Nakao, Kenji Kato, Suguru Kadowaki, Susumu Yoshida, Hironori Tsuji, Ayane Ozaki, Tomoyoshi Komiyama, Yoshikazu Ugawa, Yukio Nishimura

**Affiliations:** Neural Prosthetics Project, Tokyo Metropolitan Institute of Medical Science, Tokyo 156-8506, Japan; Department of Developmental Physiology, National Institute for Physiological Sciences, National Institutes of Natural Sciences, Okazaki 444-8585, Japan; Department of Food and Nutrition Science, Sagami Women’s University, Sagamihara 252-0383, Japan; Department of Rehabilitation Treatment, Chiba Rehabilitation Center, Chiba 266-0005, Japan; Department of Developmental Physiology, National Institute for Physiological Sciences, National Institutes of Natural Sciences, Okazaki 444-8585, Japan; Department of Developmental Physiology, National Institute for Physiological Sciences, National Institutes of Natural Sciences, Okazaki 444-8585, Japan; Department of Neurology, School of Medicine, Fukushima Medical University, Fukushima 960-1295, Japan; School of Rehabilitation Sciences, Health Sciences University of Hokkaido, Tobetsu 061-0293, Japan; Neural Prosthetics Project, Tokyo Metropolitan Institute of Medical Science, Tokyo 156-8506, Japan; Japan Society for the Promotion of Science, Tokyo 102-0083, Japan; Neural Prosthetics Project, Tokyo Metropolitan Institute of Medical Science, Tokyo 156-8506, Japan; Graduate School of Medical and Dental Sciences, Niigata University, Niigata 951-8510, Japan; Faculty of Education, Chiba University, Chiba 263-8522, Japan; Department of Human Neurophysiology, School of Medicine, Fukushima Medical University, Fukushima 960-1295, Japan; Neural Prosthetics Project, Tokyo Metropolitan Institute of Medical Science, Tokyo 156-8506, Japan; Department of Developmental Physiology, National Institute for Physiological Sciences, National Institutes of Natural Sciences, Okazaki 444-8585, Japan; Graduate School of Medical and Dental Sciences, Niigata University, Niigata 951-8510, Japan

**Keywords:** closed-loop interface, magnetic spinal stimulation, plasticity, spinal cord injury

## Abstract

Gait disturbance in individuals with spinal cord injury (SCI) at levels rostral to the lumbar locomotor centre results from disconnection between the supraspinal system and the spinal locomotor centre. Here, we present a non-invasive volition-controlled spinal stimulation paradigm that empowers paraplegic individuals to regain stepping control in their impaired legs.

Using hand muscle-controlled magnetic stimulation targeting the lumbar spinal motor circuits in the preserved lumber cord, individuals with chronic SCI achieved control of start–stop motion, step length and cadence of bilateral cyclic stepping in paralysed legs. Stimulus-induced cyclic stepping with leg muscle EMG activity was evoked in all participants with complete or incomplete SCI, regardless of the lesion site between the thoracic and lumbar spinal cord. Combining voluntary gait effort with closed-loop stimulation further enhanced leg movements. Repeated application of this closed-loop stimulation led to progressive improvement in stimulus-induced stepping and muscle responses, particularly in participants with thoracic SCI, and in stimulus-free stepping, particularly in participants with incomplete SCI. Our findings indicate that the preserved lumbar spinal motor circuit plays a crucial role in improving stimulus-induced stepping, whereas the preserved descending pathway is required for improving stimulus-free stepping.

This non-invasive closed-loop spinal stimulation paradigm bypasses the lesion site on the spinal cord and strengthens both the preserved spinal circuits and the descending pathways to allow bilateral stepping control to be regained after SCI. This approach holds great promise for SCI-related gait rehabilitation because it has the potential to lead to functional recovery. Furthermore, this approach offers a viable alternative for individuals with contraindications to invasive procedures or those who do not consent to surgical treatments.

## Teaser

Tazoe *et al.* demonstrate the feasibility of a non-invasive closed-loop spinal interface for stepping control in individuals with spinal cord injury. This interface creates an artificial pathway that compensates for the function of interrupted descending pathways and strengthens the preserved spinal circuits and descending pathways.

## Introduction

Paraplegia after spinal cord injury (SCI) rostral to the lumbar gait centre results from the interruption of neural transmission from the brain to the preserved spinal motor circuitry for gait. Hence, reformation of functional motor commands from the intact brain to the preserved spinal centre would restore volitional control of paralysed lower limbs. Closed-loop activity-dependent stimulation is a promising approach for this purpose. It creates an artificial neural connection (ANC), through which supraspinal voluntary commands can be transferred to the spinal circuitry, bypassing the lesion site with the aid of electrical stimulation decoded by a computer interface.^1-3^ Studies in animal models and in humans with damage to the descending pathways have demonstrated that an ANC is capable of restoring volitional control of functional movements in the upper^4-9^ and lower extremities.^10-13^ In addition, an ANC has the potential to strengthen synaptic connectivity in a natural pathway^14^ and is therefore expected to contribute to the functional recovery of natural voluntary motor control. However, the practical utility of closed-loop stimulation paradigms in individuals with SCI is mostly limited to invasive techniques requiring surgery to implant recording and/or stimulation electrodes.^8,13,15-17^ At present, a less invasive closed-loop paradigm is needed because it must be feasible to apply in many more individuals with SCI, irrespective of disease severity.

Here, we demonstrate the feasibility of non-invasive ANC for paraplegic humans with chronic SCI. We previously developed the non-invasive ANC technique, hand muscle-controlled percutaneous magnetic stimulation over the lumbar vertebrae, which can induce voluntarily controlled gait behaviour by activating the presumed spinal locomotor centre.^18,19^ This biomimetic activity-dependent spinal stimulation enabled chronic paraplegic individuals to control bilateral cyclic stepping, including initiating and terminating stepping and changing step length and cadence. Repetitive application of non-invasive ANC improved closed-loop controlled cyclic stepping and enhanced voluntary cyclic stepping without ANC aid. This body of evidence indicates that the non-invasive closed-loop neural interface is a feasible tool for restoring volitional movements in paralysed lower limbs and contributes to motor functional recovery in residual spinal circuits and connections between descending pathways and spinal circuits.

## Materials and methods

### Participants

The experiments were performed in 10 individuals with chronic SCI (Table 1) who provided written informed consent to participate in the present study, which was approved by the local ethics committees of Fukushima Medical University (Fukushima, Japan, approval no. 1278), the Chiba Rehabilitation Centre (Chiba, Japan, approval nos. 25-9 and 29-6), the National Institute for Physiological Sciences (Okazaki, Japan, approval no. 12B009) and the Tokyo Metropolitan Institute of Medical Science (Tokyo, Japan, approval nos. 17-2 and 25-1). All experiments were conducted at Fukushima Medical University and the Chiba Rehabilitation Centre and Tokyo Metropolitan Institute of Medical Science in accordance with the Declaration of Helsinki. Five of 10 participants (#1–#5) were enrolled in the longitudinal experimental protocol involving repeated sessions (see ‘Experimental procedures’ section). According to the International Standards for Neurological Classification of Spinal Cord Injury and the American Spinal Injury Association Impairment Scale (AIS), two participants (#1 and #2) were classified as AIS A at the thoracic level. These participants presented complete loss of sensory and motor function caudal to the spinal cord lesion level. Another participant (#3) was classified as AIS C at the thoracic level. She presented incomplete loss of motor function with palpating bilateral thigh muscles and weakly contracting ankle dorsiflexor and plantar flexor muscles on the right side. She also presented sensory abnormalities bilaterally at levels caudal to the lesion. The other two participants (#4 and #5) were classified as AIS A at the lumbar level. Although both participants presented complete loss of sensory function at levels caudal to the lesion, partial motor function in the right thigh muscle was preserved. The other five participants (#6–#10) were enrolled in single-session experiments (see Supplementary material). Among them, three participants were classified as AIS A at the cervical (#8) and thoracic (#6 and #7) levels, presenting with a complete loss of sensory and motor function caudal to the level of lesion. Participant #9 was classified as AIS B at the thoracic level, presenting complete loss of leg motor function with partial preservation of sensation in the sacral regions. Participant #10 was classified as AIS C at the lumbar level, retaining bilateral hip flexion and knee extension function without any motor function below L3 level but presenting sacral sparing.

**Table 1:** Participant profile and number of testing sessions and stimulus pulses with non-invasive artificial neural connection

Longitudinal experiments					
Participants	#1	#2	#3	#4	#5
Age, years	42	54	19	27	21
Sex	Male	Male	Female	Male	Male
Time since injury, months	28	20	22	33	7
Lesion level^a^	Th11	Th3	Th8	L1	L1
**AIS**					
Before	A	A	C	A	A
After	A	A	C	A	A
**LEMS** ^b^ **(L/R)**					
Before	0/0	0/0	0/11	0/2	0/1
After	0/0	0/0	2/11	0/4	3/4
**Pin prick sensory score** ^c^ **(L/R)**					
Before	34/34	18/18	34/43	38/38	38/38
After	34/34	18/18	35/43	38/38	38/38
**Light touch sensory score** ^c^ **(L/R)**					
Before	34/34	18/18	30/41	38/38	38/38
After	34/34	18/18	31/41	38/38	38/38
Number of sessions	6	13	17	19	18
**Session interval, days**					
Average	11.2 ± 9.4	26.8 ± 19.5	19.3 ± 28.8	14.6 ± 18.9	18.1 ± 30.7
Maximum	28	63	112	85	127
Minimum	7	6	6	5	1
**Number of stimulations**					
Total	17 784	56 712	45 704	72 377	45 867
Average per session	3417 ± 714	4363 ± 2234	2566 ± 1371	3806 ± 1695	2548 ± 1618

Single-session experiments					
Participants	#6	#7	#8	#9	#10
Age, years	40	45	27	29	29
Sex	Female	Female	Female	Female	Male
Time since injury, months	85	204	83	48	77
Lesion level^a^	Th10	Th1	C7	Th3	L2
AIS	A	A	A	B	C
LEMS^b^ (L/R)	0/0	0/0	0/0	0/0	9/10
Pin prick sensory score^c^ (L/R)	36/36	20/22	12/11	32/50	45/44
Light touch sensory score^c^ (L/R)	35/35	21/22	12/11	32/51	44/43

For long-term experiments, AIS, LEMS and sensory scores were obtained before the first session and after the last session (only for Participant #3, the data were obtained before the first session and after the 17th session). For Participant #3, session and stimulation data were collected before 3 years of a long break (i.e. until the 17th session) for statistical comparison. All the sessions are presented in Supplementary Table 1. The stimulus data were collected from all non-invasive artificial neural connection (ANC) trials in which >30 stimulus pulses were applied. Averaged data are presented with standard deviations. AIS = American Spinal Injury Association Scale; LEMS = Leg Extremity Motor Score.

^a^The lesion level indicates the most rostral spinal segment showing sensory and/or motor impairment.

^b^LEMS on each side is a maximum of 25.

^c^Score on each side is a maximum of 56.

Before enrolling in the present study, all participants had received regular rehabilitation by physiotherapists for at least half a year. It was confirmed that regular rehabilitation did not result in further improvement in the AIS in any of the participants.

For Participants #2–#5 in the longitudinal experimental protocol, exploratory data were collected for ∼40 weeks. The experimental 1-day sessions were repeated every 1–2 weeks, with some exceptional break periods for participants’ personal reasons. Participant #1 repeated the sessions for 8 weeks until he was unable to rejoin the study (Supplementary Table 1). Only Participant #3 returned for an extra two sessions after 3 years (IRB approval no. 29-6; Supplementary Table 1). The remaining five participants were enrolled in single-session experiments: two participants (#6 and #7; IRB approval nos. 17-2 and 25-1) underwent two sessions, and the other three participants (#8, #9 and #10; IRB approval no. 17-2; Supplementary material ‘Methods’ section) underwent a single session.

### Experimental procedures

**Figure 1:**
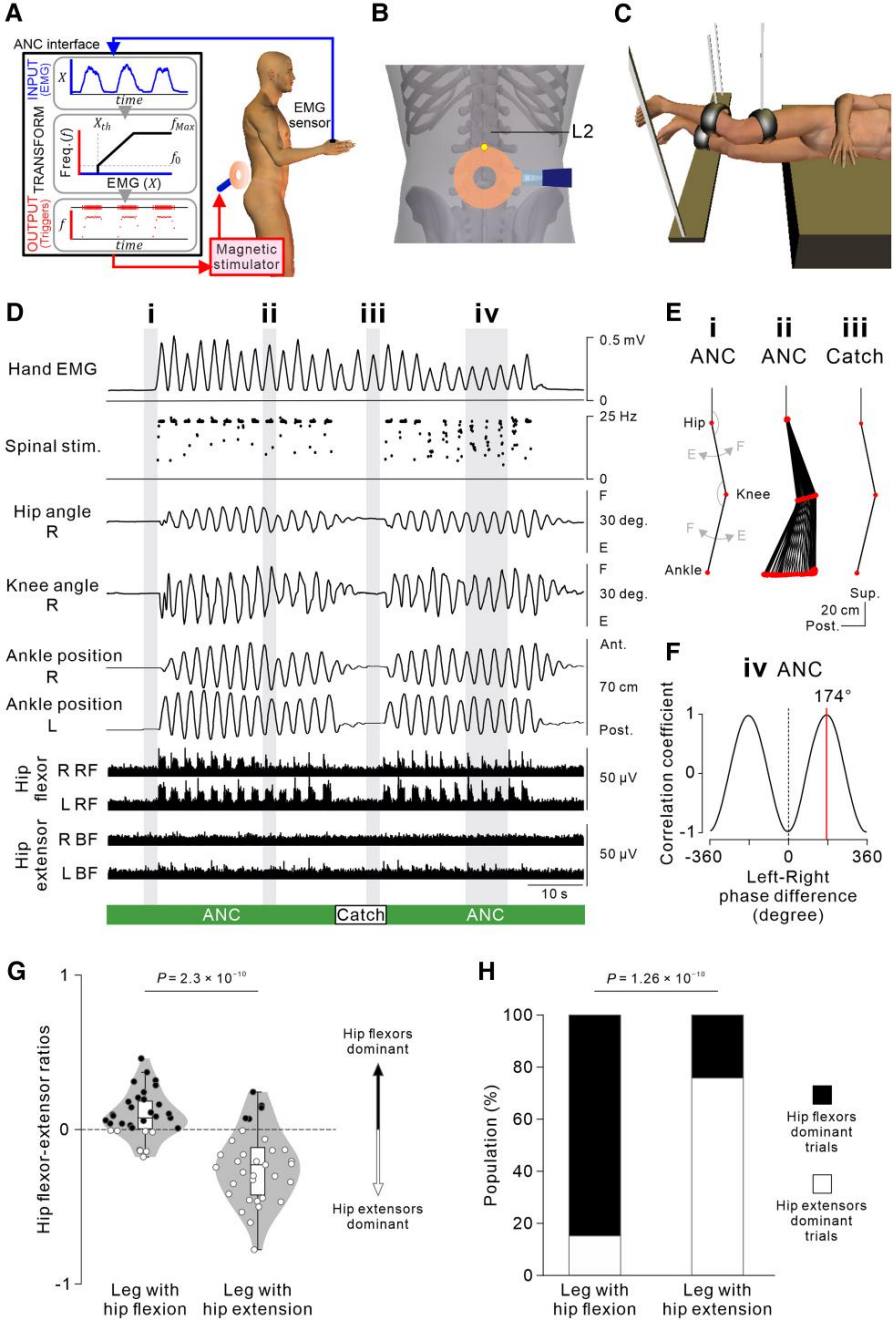
**Hand EMG-controlled cyclic leg stepping using the non-invasive artificial neural connection (ANC) interface.** (**A**) Scheme of the non-invasive ANC interface. Hand EMG-controlled magnetic stimulation was delivered over the lumbar vertebrae via a computer interface. (**B**) Stimulus coil location. The upper edge of the round coil was set at an intervertebral space between L2 and L3 (yellow dot). (**C**) Two-leg suspension system. Bilateral shanks and thighs were securely placed on braces suspended from the ceiling by ropes. This posture allowed participants to perform bilateral cyclic stepping without constraints from gravity. (**D**) Representative example of hand EMG-controlled bilateral cyclic stepping using the non-invasive ANC interface in Participant #2 with complete SCI at the Th3 level [fourth session (Day 29)]. The green and white bars on the bottom show the periods during which the ANC interface was turned on (ANC) and off (Catch), respectively. Magnetic stimulation was delivered at 60% of the maximum stimulator intensity. Note that stimulus artefacts were flattened out in the bottom four EMG traces. (**E**) Stick pictures showing the right leg kinematics in the grey hatched time windows in **D** before (**i**) and during hand gripping with the ANC interface (**ii**) and without the ANC interface (‘catch cycle’; **iii**). (**F**) Cross-correlogram of left and right leg movements at the anterior–posterior axis in three successive cycles (**iv** in **D**). The red vertical line indicates the phase difference between the left and right leg stepping movements. These data were obtained from Supplementary Video 1. (**G**) Hip flexor–extensor ratios estimated by the muscle activity of bilateral iliopsoas (Iliop), gluteus maximus (Glut), rectus femoris (RF) and biceps femoris (BF) muscles obtained in a total of 33 ANC trials showing bilateral alternate leg stepping in five participants (#6–#10). Box and violin plots represent data variation and distribution for each leg where magnetic spinal stimulation induced hip flexion (*left*) and extension (*right*), respectively (*n* = 33, *t* = −9.073, *P* = 2.3 × 10^−10^; two-tailed paired *t*-test). Black and white circles indicate that the estimated dominant muscle activity was hip flexors and extensors, respectively. (**H**) Population of trials in which hip flexors (black) or hip extensors (white) were predominant at each leg (*n* = 33, *P* = 1.26 × 10^−10^; Fisher’s exact probability test).

The experimental procedures were similar to those described previously.^18-21^ Briefly, to achieve an ANC that sends voluntary commands to the lumbar motor circuits and bypasses a spinal cord lesion via a non-invasive approach, EMG signals from the first dorsal interosseous muscle (‘Hand EMG’) were converted to stimulus pulses (Fig. 1A). These pulses were used to trigger magnetic stimuli delivered over the lumbar vertebrae (Fig. 1B). Hand EMG (the first trace in Fig. 1D) during hand gripping was used to control the ANC (see details in the Supplementary material, ‘Methods’ section). The participants were in a semiprone position on a comfortable bed (Fig. 1C). The lower limbs were suspended by wires to keep the participants relaxed. This apparatus supported low-friction movement of the legs such that the participant could readily induce leg movements in a horizontal plane. A mirror was set in front of the participant to provide visual feedback on their own leg movements. An experimental session consisted of 4–35 trials. One trial consisted of one of the three types of cyclic stepping tasks. In the first task, the participants were instructed voluntarily to perform bilateral cyclic stepping in the semiprone posture without using the non-invasive ANC (‘Vol.’). In the second task, the participants were instructed to perform bilateral cyclic stepping using the non-invasive ANC controlled by hand muscle contraction in relaxed lower-limb muscle conditions (‘ANC’). The participants were instructed to generate bilateral alternate cyclic leg stepping by performing cyclic hand gripping. The participants controlled the strength and rhythm of hand gripping using visual feedback of the leg movements to achieve alternating leg movements (forwards in one leg and backwards in the other) during the terms of stimulus trains. Third, the participants performed bilateral cyclic stepping using the non-invasive ANC in association with voluntary effort to move their legs (‘ANC + Vol.’). In all the tasks, the participants tried to perform alternate stepping as large as possible. In one trial, the participants performed rhythmic hand gripping for ∼40 s [41.4 ± 31.7 s (6.2–210.1 s)], which allowed us to collect data from ∼20 stepping cycles [19.8 ± 14.3 cycles (4–87 cycles)]. In one session, the participants performed any of the three or all tasks multiple times randomly with some break time [1.97 ± 2.65 min (11 s–29.58 min)]. Because of participant fatigue, the total number of trials in one session was highly variable across sessions and participants (Supplementary Table 1).

In some trials, we arranged specific protocols and gave the participants particular instructions for the stepping tasks to address the following specific research questions. To confirm the stimulation effect of the non-invasive ANC, the ANC interface was unpredictably turned off such that stimulus triggers could not be delivered for a certain period although the participants were maintaining hand gripping (‘Catch cycles’). Magnetic spinal stimulation would have been necessary to generate bilateral alternating steps in these participants, hence we expected no stepping to be observed during ‘Catch cycles’. In some other trials, to verify the controllability of ANC-controlled cyclic stepping, we specifically instructed the participants to change the step cycle duration from long (‘long’) to short (‘short’) by changing the hand-gripping duration. We measured the duration of hand EMG activity, number of trigger pulses, step length, ankle trajectory length and step cadence in these experimental trials to demonstrate that the participants were able to control the step length in ANC-controlled bilateral alternating leg movement.

To investigate the effects of successive non-invasive ANC trials on the same day, ANC-controlled cyclic stepping (‘ANC’) was performed three times at intervals of 5 min. We expected that ANC-controlled stepping would immediately improve in every trial and that their summation would occur because repeated sessions might cause plastic changes in the stimulated residual spinal motor circuits. Stimulus parameters such as coil position, intensity and temporal profiles for triggers were fixed within a participant in this experiment. We also investigated the effect of voluntary leg movement effort on ANC-controlled cyclic stepping. If the participants preserved even slight voluntary descending drives, ANC-controlled stepping might have improved owing to greater leg muscle activation produced by summative inputs of spinal stimulation and voluntary descending drives to the lumbar spinal motor circuits. The participants tried to perform ANC-controlled cyclic stepping without and with voluntary effort for leg stepping in one trial. Catch cycles (with the ANC interface off) were also included in the same trial. To quantify the magnitude of leg stepping, the participants also performed trials of each condition separately to record at least three continuous cycles of steady stepping. In addition, to investigate whether the combination of the non-invasive ANC and voluntary effort had an immediate after-effect on stimulation-free natural voluntary cyclic stepping (‘Vol.’), we measured the magnitude of volitional cyclic stepping immediately after a single trial of ANC-controlled cyclic stepping with voluntary effort (‘ANC + Vol.’). In this trial, the spared lumbar motor circuits are repeatedly activated by spinal stimulation together while receiving residual descending drives, which might induce plastic neuromodulation in the residual descending motor pathways and improve voluntary leg stepping movements. We repeated this measurement three times consecutively. The stimulus parameters were also fixed in all three trials. These experimental trials were conducted in an exploratory manner after the participants had already completed several ANC sessions (Supplementary material, ‘Methods’ section).

## Results

### Control of bilateral leg stepping using closed-loop magnetic spinal stimulation

To establish a non-invasive ANC interface that transfers voluntary commands to preserved lumbar spinal motor circuits while bypassing the spinal cord lesion, we instructed the participants to perform repetitive hand gripping at their own rhythm to manipulate the temporal profiles of magnetic stimulation (i.e. its onset, offset and rhythm). By observing their own on-time bilateral leg movements through a mirror, the participants modulated the frequency of magnetic stimulation targeting the lumbar motor circuits by voluntarily changing their hand muscle activity to control the bilateral cyclic leg stepping through the interface. Figure 1D shows a typical example of ANC-controlled bilateral cyclic stepping in Participant #2, with complete loss of lower-limb sensorimotor functions owing to a lesion at the third thoracic spinal cord level (see also Supplementary Video 1). When the participant relaxed their hand, leg movement was not induced [Fig. 1D(i) and E(i)] because the level of hand EMG activity in the first dorsal interosseous was below the threshold for delivering spinal stimulation. The initiation of hand gripping immediately induced bilateral leg stepping, in which the left and right paralysed legs moved in opposite directions. With the support of pendulum motion, alternating cyclic stepping was maintained during repetitive hand gripping [Fig. 1D(ii and iv), E(ii) and F(iv)] and gradually stopped when the participant stopped hand gripping (Fig. 1D). The leg muscles showed rhythmic burst-like activities coincident with the magnetic stimulation triggers. Although the left and right hip joint motions were opposite, a flexor-dominant activation pattern was observed on both sides. Interestingly, the left biceps femoris (BF) muscle showed weak rhythmic activation, probably contributing to hip extension movement on the left side, whereas the right BF muscle remained inactive, because the right side was undergoing hip flexion (Fig. 1D, bottom four traces). Given that we recorded the hip flexor and extensor EMGs only from the thigh muscles in the longitudinal experimental protocol, we examined separately whether muscle activity was also induced in the pelvic hip flexor [iliopsoas (Iliop)] and extensor muscles [gluteus maximus (Glut); Supplementary Fig. 1] in addition to the rectus femoris (RF) and BF muscles, which is consistent with hip joint motion, using a non-invasive ANC interface. We tested five participants (#6–#10) with chronic SCI (see Supplementary material, ‘Methods’ section) in the single-session experiments and observed asymmetric activation in the hip flexor group (Iliop and RF) and hip extensor group (Glut and BF) (Supplementary Fig. 2). The predominant activation across hip flexor group and extensor group was opposite between legs (Fig. 1G) and corresponded to the observed hip joint motions during hand EMG-controlled alternate stepping (Fig. 1H).

The magnitude of leg stepping was often asymmetric across sides, with larger movement typically observed in the leg exhibiting hip flexion during stimulus bursts. However, there was no significant corelation in the leg stepping magnitude across sides (Supplementary material, ‘Results’ section). To rule out the possibility that smaller-sized leg stepping movement on one side was merely a passive counter-motion in response to the contralateral larger-sized leg stepping movement, we further confirmed that each of hip flexion and extension movements during stimulus bursts were preserved even when the contralateral leg motion was mechanically restricted (Supplementary Fig. 3).

To confirm the bilateral cyclic stepping induction via the non-invasive ANC interface, the spinal stimulation was halted unpredictably during the catch cycles for several seconds (Fig. 1D, white bar). During the catch cycles, hand gripping failed to evoke cyclic stepping or leg muscle activities [Fig. 1D(iii) and E(iii)]. When magnetic spinal stimulation was delivered continuously at a constant frequency of 25 Hz without using the ANC interface, bilateral alternative cyclic stepping was not induced. Because leg movements did not oscillate during continuous stimulation, the trajectory lengths of the bilateral leg were shorter than those of hand EMG-controlled stimulation through the ANC interface (Supplementary Fig. 4).

**Figure 2:**
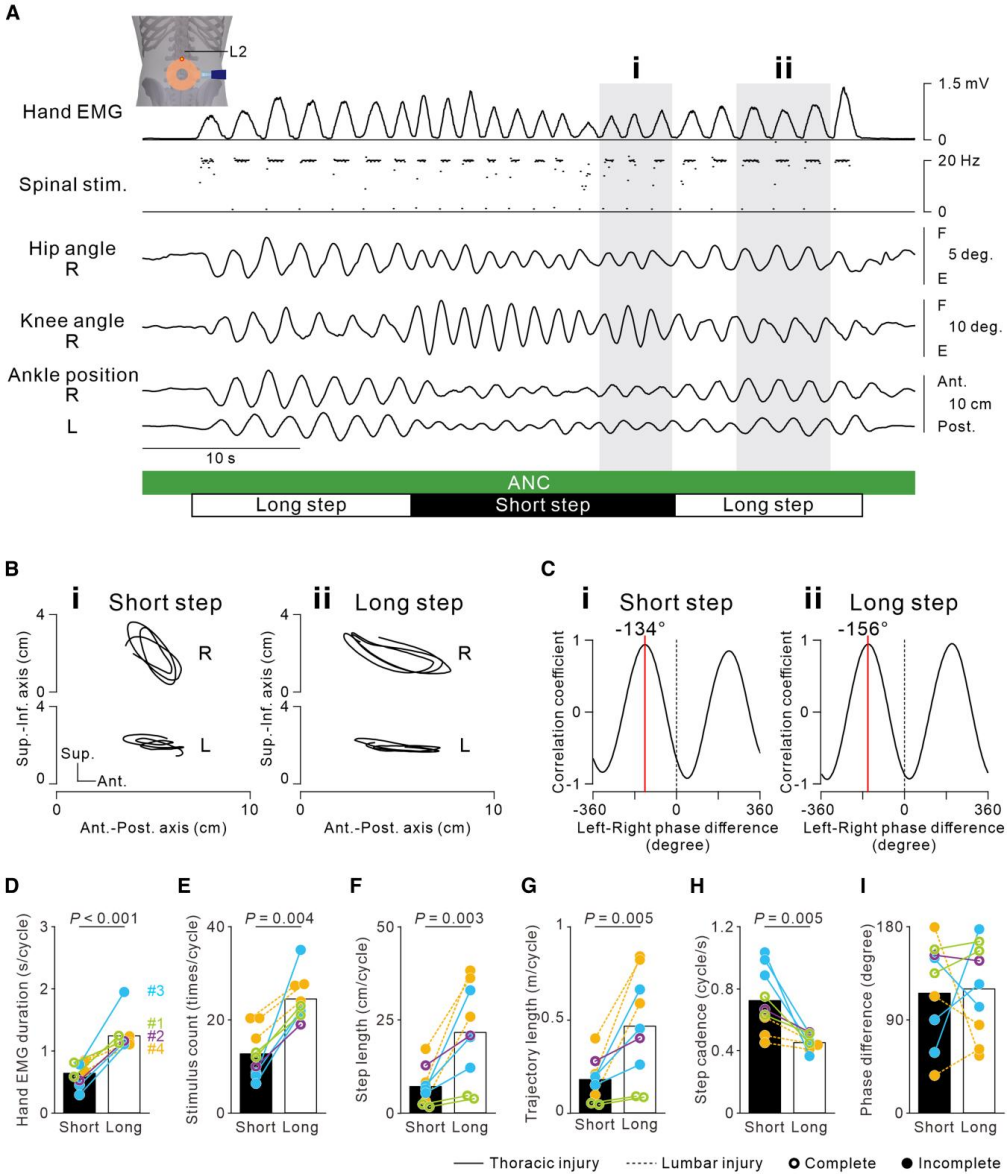
**Control of step length using the non-invasive artificial neural connection (ANC) interface.** (**A**) A participant manipulated his hand gripping duration to control his step length through the ANC interface. The white and black horizontal bars represent the periods during which the participant gripped his hand for short and long durations, respectively. The grey hatched areas (**i** and **ii**) correspond to the time windows for the data presented in **B** and **C**. These data were obtained from Supplementary Video 2 of Participant #1 with complete spinal cord injury (SCI) at the Th11 level during the sixth session (Day 57). Spinal stimulation was delivered to the L2–L3 intervertebral region at an intensity of 65% of the maximum stimulator output. (**B** and **C**) Bilateral ankle trajectories (**B**) and cross-correlogram of left and right leg movements at the anterior-posterior axis (**C**) in three successive cycles. Left (**i**) and right (**ii**) data were obtained in the time windows ‘**i**’ and ‘**ii**’, respectively, in **A**. (**D**–**I**) Circles and bars represent individual data and mean data, respectively, for hand EMG duration (**D**), stimulus count (**E**), step length (**F**), ankle trajectory length (**G**), step cadence (**H**) and phase difference between left and right leg movements at the anterior–posterior axis (**I**). Note that the absolute values of the phase differences were transformed to a range of 0°–180°. For each participant, these data were obtained during the sixth (#1), seventh (#2), 18th and 19th (#3) and fifth sessions (#4). Two-tailed paired *t*-tests (**D** and **F**–**I**, *n* = 9) or Wilcoxon signed rank tests (**E**, *n* = 9) were performed to compare the short- and long-step conditions.

To further verify the controllability of ANC-controlled bilateral stepping, the participants were instructed to modulate the step length by changing the duration of hand EMG bursts via the non-invasive ANC interface. The step lengths during the long hand grip period were longer than those during the short hand grip period (Fig. 2 and Supplementary Video 2). The cadence of cyclic stepping was also controllable in association with modulation of the step length (Fig. 2H) while maintaining left–right leg coordination (*t* = −0.0324, *P* = 0.975, two-tailed paired *t*-test; Fig. 2C and I). Thus, using their own hand muscle activity, the participants with SCI were able to initiate and terminate bilateral cyclic stepping and control step length and cadence through the non-invasive ANC interface. Regardless of the presence of spinal cord lesions between the thoracic and lumbar levels, the participants with either complete or incomplete loss of lower-limb sensorimotor function were able to control bilateral cyclic stepping (Fig. 2D–I).

### Immediate improvement in ANC-controlled cyclic stepping by repetition

**Figure 3:**
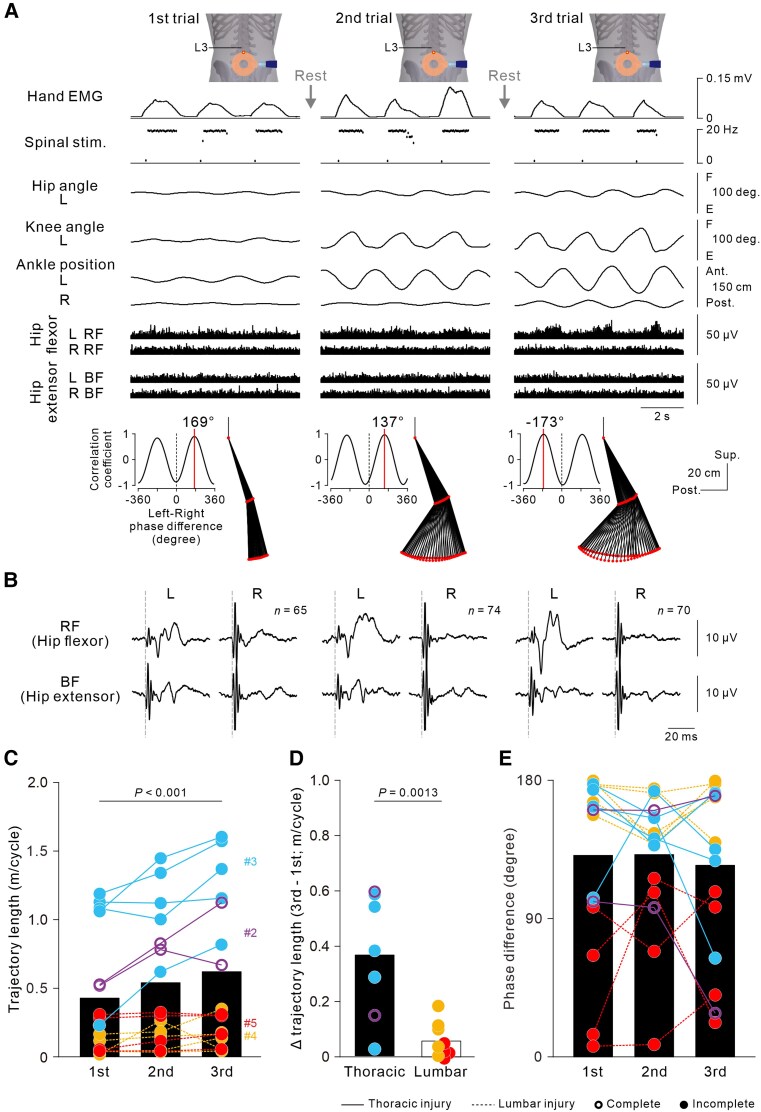
**Improvement in artificial neural connection (ANC)-controlled stepping with repeated ANC interface use within 1 day.** (**A**) Representative examples of a series of ANC-controlled cyclic stepping data for three consecutive trials in Participant #3 with incomplete spinal cord injury (SCI) at the Th8 level obtained during the eighth session (Day 65). Note that stimulus artefacts were flattened out in the bottom four EMG traces. Stick pictures and cross-correlograms show a cycle of left leg kinematics and the phase difference between left and right leg movements in the above three continuous cycles. Spinal stimulation was applied over the L3–L4 intervertebral region at an intensity of 60% of the maximum stimulator output. The data were obtained from Supplementary Video 3. (**B**) Stimulus-triggered averaging EMGs in the bilateral hip flexor and extensor muscles in three successive cycles. Here, the stimulus artefacts were not flattened, to indicate stimulus onset. (**C**) Ankle trajectory length. The circles represent the data of individual participants in each series. A series of trials was repeatedly performed over a few days by the four participants (two, five, five and four times for Participants #2, #3, #4 and #5, respectively). For each participant, the experiments were performed in the sixth and 11th sessions (#2), eighth and 13th–16th sessions (#3), third and sixth–ninth sessions (#4), and fifth and 10th–12th sessions (#5). The black bars indicate the grand average of all series in all participants [*n* = 16, *F*(2,30) = 9.597, *P* < 0.001; one-way ANOVA followed by multiple comparisons with Bonferroni correction]. (**D**) Differences in trajectory length between the first and third trials. The bars and circles represent the average and each series of data in the thoracic (*n* = 7 in Participants #2 and #3) and lumbar SCI groups (*n* = 9 in Participants #4 and #5), respectively. A two-tailed unpaired *t*-test was performed to compare groups. (**E**) The phase differences between the left and right leg movements. Note that the absolute values of the phase differences were transformed to a range of 0°–180°.

To investigate the effects of the repetitive use of the non-invasive ANC interface, the participants performed three successive trials of ANC-controlled cyclic stepping while the lower limbs were fully relaxed. Parameters of leg kinematics, such as the hip and knee joint angles and trajectories of the knees and ankles, gradually and progressively increased (Fig. 3A and Supplementary Video 3), even though the participants performed identical hand gripping for consistent spinal stimulation throughout the three trials (Supplementary Table 2). We also observed a progressive increase in the left RF muscle activity, which drove left hip joint movements (Fig. 3A). Although it was not obvious in the raw EMG traces, the stimulus-triggered averaging technique revealed small muscle activations concealed in the background noise. On the left side, the stimulus-evoked muscle response in the RF muscle was selectively enhanced by repeated trials. In contrast, the activity in the right BF, the agonist of the observed right hip extension during the stimulus phase, did not show a comparable enhancement (Fig. 3B). Population-level analysis revealed that the overall average foot trajectory of the four participants improved significantly in length [*F*(2,30) = 9.597, *P* < 0.001; one-way ANOVA; black bars in Fig. 3C]. The degree of improvement was significantly greater in the thoracic SCI group than in the lumbar SCI group (*t* = 4.006, *P* = 0.0013; two-tailed unpaired *t*-test; Fig. 3D). The phase differences between the left and right leg movements did not change significantly across the three consecutive trials [*F*(2,30) = 0.265, *P* = 0.769; one-way ANOVA; Fig. 3E].

### Voluntary effort boosts ANC-controlled cyclic stepping

**Figure 4:**
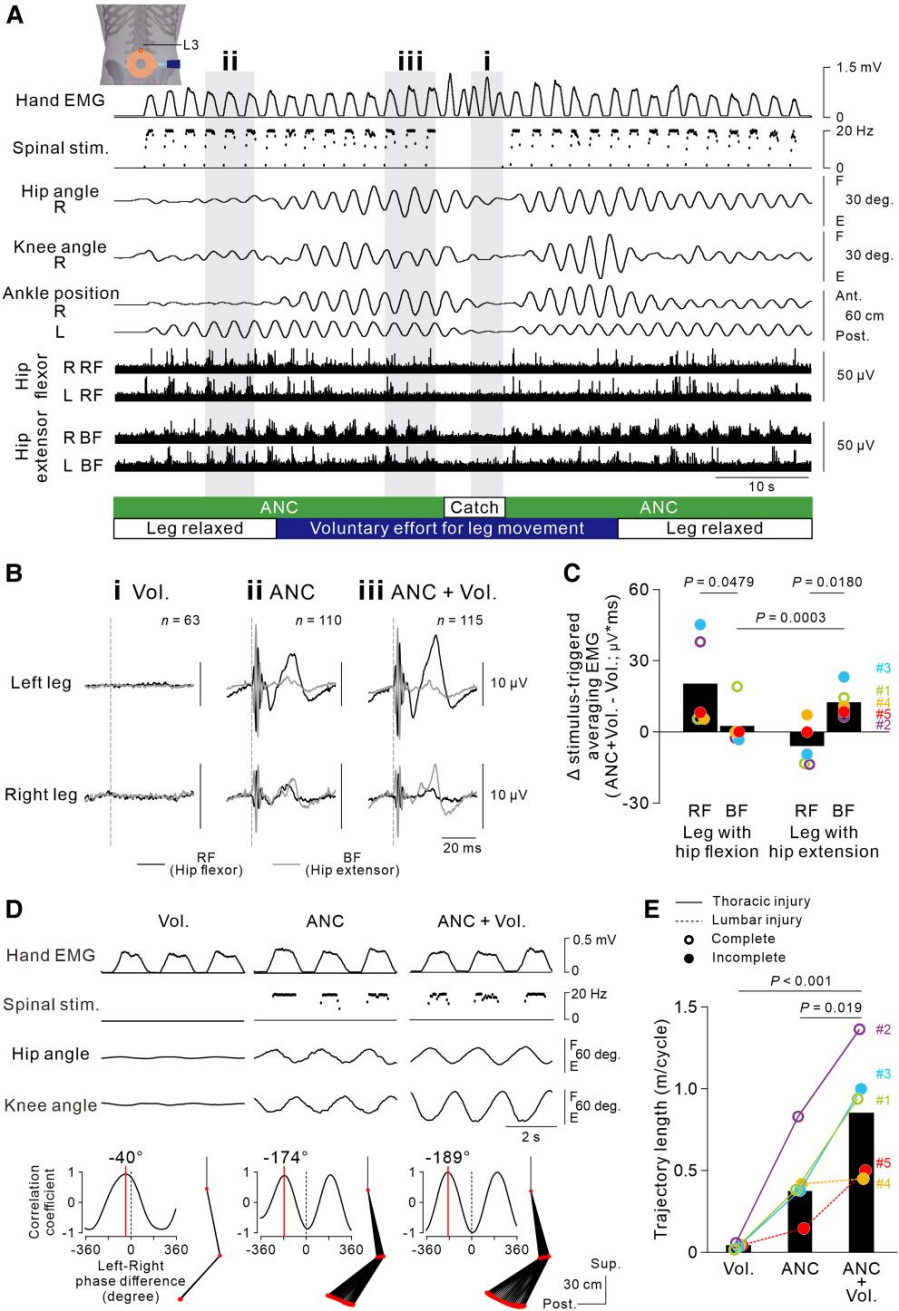
**Effect of voluntary effort on artificial neural connection (ANC)-controlled cyclic stepping.** (**A**) Representative example of ANC-controlled cyclic stepping without and with voluntary effort in Participant #3 with incomplete spinal cord injury (SCI) at the Th8 level. The green–white and blue–white bars below the traces represent the times when the ANC interface was turned on or off (catch cycle) and when voluntary effort was made for leg movements, respectively. The grey hatched areas (**i**–**iii**) correspond to the time windows for the EMG data presented in **B**. Note that stimulus artefacts were flattened out in the bottom four EMG traces. The data were obtained from Supplementary Video 4. (**B**) Stimulus-triggered averaging EMGs in the bilateral hip flexor and extensor muscles in three successive cycles (**i**–**iii** in **A**). In the time window ‘**i**’, the EMG was averaged with reference to the virtual output events of the ANC interface. The stimulus artefacts in the time windows ‘**ii**’ and ‘**iii**’ were not flattened, to indicate stimulus onset. (**C**) Difference in the stimulus-triggered averaging EMGs between conditions of ANC-controlled stepping without (‘ANC’) and with voluntary effort (‘ANC + Vol.’). Black bars indicate the average of all participants (*n* = 5; fixed effect of MUSCLE, χ^2^ = 8.8120, *P* = 0.002993; fixed effect of LEG, χ^2^ = 2.3524, *P* = 0.125092; interaction of MUSCLE and LEG, χ^2^ = 15.7991, *P* = 7.044 × 10^−5^, generalized linear mixed effect model followed by Bonferroni corrected *t*-tests). (**D**) Typical recordings of stationary cycles of stepping performance in Participant #3. From *left* to *right*, the conditions were voluntary cyclic stepping without stimulation (‘Vol.’), ‘ANC’ and ‘ANC + Vol.’. Stick pictures and cross-correlograms show a cycle of left leg kinematics and the phase difference between left and right leg movements in the above three continuous cycles. Spinal stimulation was applied over the L3–L4 intervertebral region at an intensity of 70% of the maximum stimulator output. (**E**) Ankle trajectory lengths of all participants. The circles represent individual participants. These data were obtained during the sixth, 13th, first, fourth and fifth sessions for Participants #1, #2, #3, #4 and #5, respectively. The black bars indicate the averages of all participants [*n* = 5, *F*(2,8) = 19.695, *P* = 0.001; one-way ANOVA followed by multiple comparisons with Bonferroni corrections].

To investigate whether ANC-controlled cyclic stepping can be boosted by voluntary effort, we asked participants to make a voluntary effort to try to move their legs during ANC-controlled cyclic stepping. Although the ANC interface was turned off, Participant #3, who had incomplete SCI at the thoracic level, could voluntarily perform a small movement only in the right ankle joint and toe; ANC-controlled bilateral cyclic stepping was improved by the addition of even such a weak voluntary drive (Fig. 4A and D and Supplementary Video 4). Stimulus-triggered averaging EMGs revealed that magnetic stimulation induced a small muscle response in the bilateral RF and BF muscles. The stimulus-evoked responses in the left RF and the right BF were clearly observed during ANC-controlled stepping in Participant #3 [‘ANC’; Fig. 4B(ii)], but this pattern was not consistently evident across other participants (Supplementary Fig. 5). Importantly, in the condition of ANC and voluntary effort (‘ANC + Vol.’), selective enhancement of muscle response was observed in the muscles serving for the observed hip joint motions (i.e. RF for the leg with hip flexion, BF for the with hip extension) [‘ANC + Vol.’; Fig. 4B(iii) and C]. In contrast, no clear EMG signals were observed in either muscle under voluntary effort alone [‘Vol.’; Fig. 4B(i)]. The voluntary effort also boosted ANC-controlled stepping even in participants with complete loss of sensorimotor function who were not able to produce any visible leg movements by voluntary effort alone (Participants #1 and #2). This voluntary effort boost was effective irrespective of the lesion level (Fig. 4E). The average ankle trajectory length was significantly greater with the combination of voluntary effort and the ANC interface than with voluntary effort alone (*t* = 6.245, *P* < 0.001, Bonferroni’s corrected *t*-test) or the ANC alone (*t* = 3.666, *P* = 0.019, Bonferroni’s corrected *t*-test; Fig. 4E) in all the participants.

### Immediate improvement in natural stepping after non-invasive ANC use combined with voluntary effort

**Figure 5:**
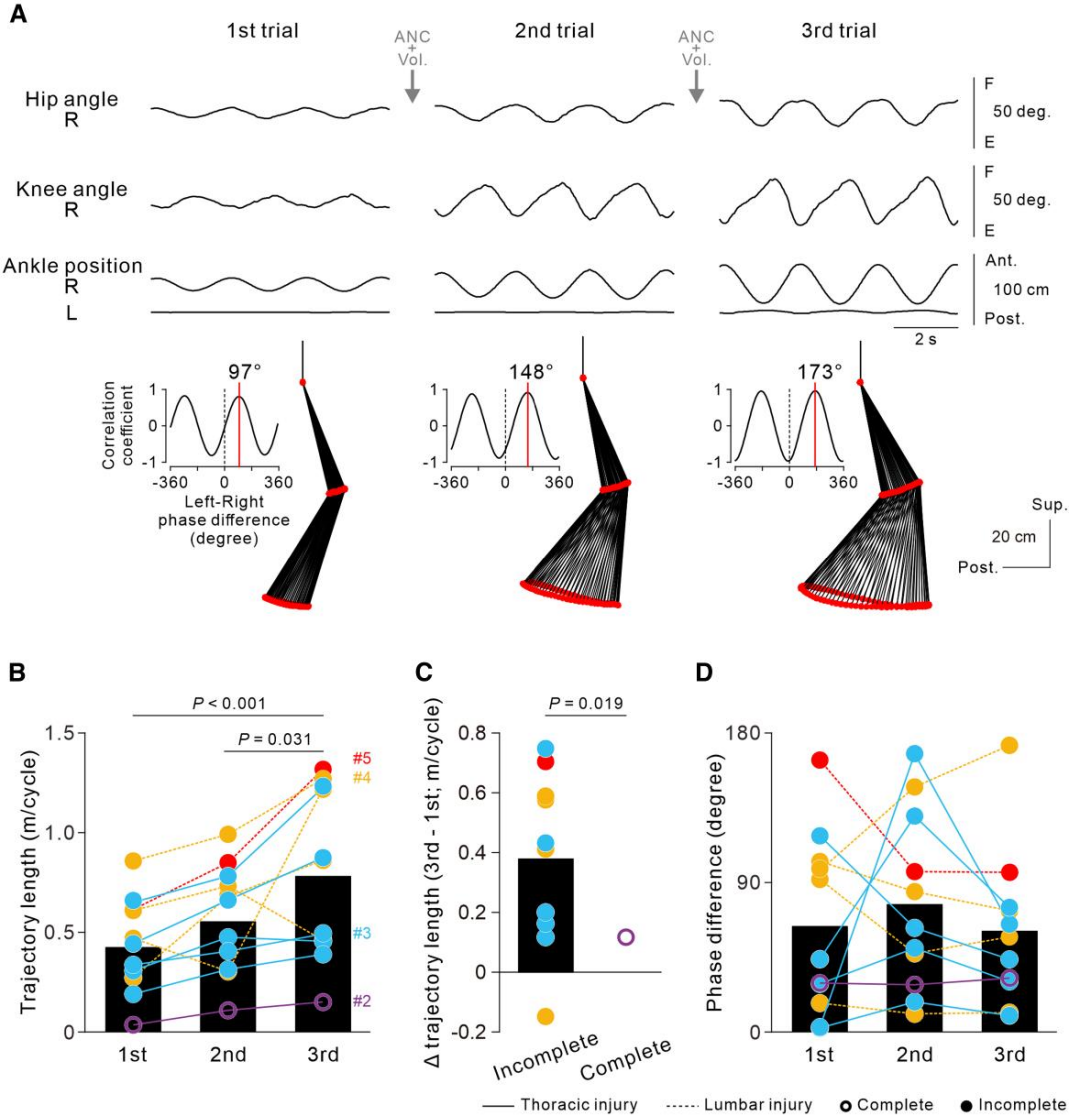
**Immediate improvement in stimulation-free voluntary stepping with artificial neural connection (ANC) intervention with voluntary effort.** (**A**) Representative examples of stimulation-free, natural voluntary stepping after the ANC intervention with voluntary effort in Participant #4 with incomplete spinal cord injury (SCI) at the L1 level obtained during the 11th session (Day 115) from Supplementary Video 5. Stick pictures and cross-correlograms show a cycle of right leg kinematics and the phase difference between left and right leg movements in the above three continuous cycles. The stimulus intensity was fixed at 60% of the maximum output, and stimulation was applied over the Th12–L1 intervertebral region. (**B**) The circles show the data of individual participants after each of three successive intervention trials. This experiment was conducted once for Participants #2 and #5, and five and four times for Participants #3 and #4, respectively. For each participant, this experiment was performed in the 11th (#2), sixth, seventh and ninth–11th (#3), fifth and 10th–12th (#4) and 13th sessions (#5). The black bars indicate the grand average of all intervention trials for Participants #2–#5 [*n* = 11, *F*(1.313,13.132) = 10.717, *ε* = 0.657, *P* = 0.004; one-way ANOVA followed by multiple comparisons with Bonferroni corrections]. (**C**) Differences in trajectory length between the first and third trials. The bars and circles represent the average and each series of data in the incomplete (*n* = 10 in Participants #3, #4 and #5) and complete SCI groups (*n* = 1 in Participant #2), respectively. A two-tailed one-sample *t*-test was performed to compare groups. (**D**) The phase differences between the left and right leg movements. Note that the absolute values of the phase differences were transformed to a range of 0°–180°.

Here, we studied whether non-invasive ANC interface use associated with voluntary effort could immediately improve stimulation-free natural stepping performance. We measured the stepping trajectory length while participants performed bilateral cyclic stepping without the ANC for ∼30 s. Measurements were obtained three times immediately after the 2 min intervention trial with the ANC combined with voluntary effort (‘ANC + Vol.’). The volitional stimulation-free stepping improved progressively over the course of successive intervention trials (Fig. 5). In Participant #4, who had incomplete loss of lower-limb sensorimotor function, the leg joint movements in the second and third trials were greater than those in the first trial (Fig. 5A and Supplementary Video 5). Volitional cyclic stepping was improved in participants with lumbar SCI and in those with thoracic SCI [foot trajectory length: *F*(1.313,13.132) = 10.717, *ε* = 0.657, *P* = 0.004, one-way ANOVA; Fig. 5B], although the improvement was significantly greater in participants with incomplete SCI than in one with complete SCI (*t* = 2.868, *P* = 0.019, two-tailed one-sample *t*-test; Fig. 5C). Consistent changes in the phase differences between left and right leg movements were not observed among the three trials [*F*(2,20) = 0.601, *P* = 0.558; one-way ANOVA; Fig. 5D]. Visual inspection of the recorded EMGs did not reveal any muscle activity around the knee or hip joints in any of the participants.

### Functional recovery through long-term non-invasive ANC application

To investigate whether long-term application of the non-invasive ANC interface can induce functional recovery of leg stepping, we continuously tracked the size of cyclic steps with and without the ANC in every testing session over months. Initially, we measured the ankle trajectory length during voluntary cyclic stepping without the ANC in the first trial of voluntary cyclic stepping without stimulation (‘Vol.’). Subsequently, we measured the trajectory length during cyclic stepping with the non-invasive ANC alone (‘ANC’ trials) in the first trial of ANC-controlled cyclic stepping, in which the stimulation targeted the most effective intervertebral region to induce bilateral alternating leg stepping in each session. Because only five cycles were recorded in some sessions, for fair intersession comparisons, we specifically measured the largest three successive steps in the first five cycles.

**Figure 6:**
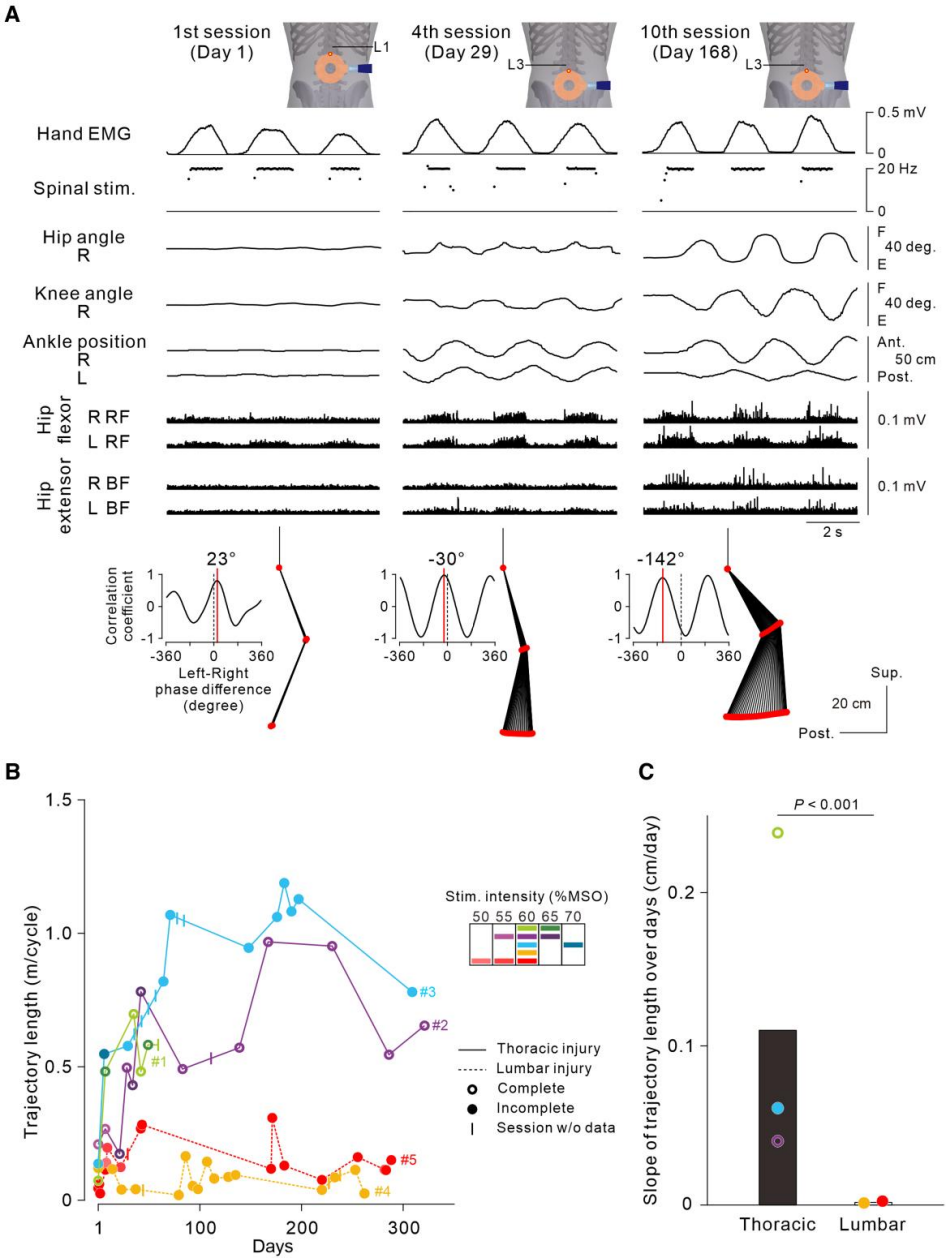
**Improvement in artificial neural connection (ANC)-controlled cyclic stepping over months.** (**A**) Representative examples from the first trial of ANC-controlled cyclic stepping in each session in Participant #2 with complete thoracic spinal cord injury (SCI). The stimulus intensity was 55% (L1–L2) on Day 1, 60% (L3–L4) on Day 29 and 60% (L3–L4) on Day 168. The arrangement of each trace, stick plot and cross-correlogram was the same as that in Fig. 3A. (**B**) Time courses of trajectory length in all five participants. The types of circles and lines indicate the characteristics of the SCI (complete or incomplete, thoracic or lumbar injury). (**C**) Slope of trajectory length increment over days. The bars and circles represent the average and individual data, respectively, for the thoracic and lumbar SCI groups. The significant difference among groups was demonstrated by analysis of covariance performed on the dataset in **B** with a generalized linear mixed-effects model (interaction between Day and Group, χ^2^ = 155.5685, *P* = 7.957 × 10^−5^, *n* = 6, 12, 11, 17 and 16 for Participants #1, #2, #3, #4 and #5, respectively).

In Participant #2, who experienced complete injury at the thoracic level, long-term application of the non-invasive ANC interface progressively increased ANC-controlled steps and EMG activity around the hip joints (Fig. 6A and Supplementary Video 6). In three participants with thoracic SCI, the ankle trajectory length gradually increased regardless of complete or incomplete loss of lower-limb sensorimotor function (Participants #1, #2 and #3 in Fig. 6B). In contrast, participants with lumbar lesions showed no clear progressive improvement in cyclic stepping (Participants #4 and #5 in Fig. 6B). The difference in the effectiveness of the ANC between the two groups of subjects (those with thoracic and lumbar lesions) was supported by the significant difference in the slope of the ankle trajectory length over days (χ^2^ = 155.5685, *P* = 7.957 × 10^−5^, analysis of covariance with generalized linear mixed-effects model; Fig. 6C) despite the lack of a difference in the interval between ANC sessions or the number of stimulus pulses per session (*n* = 68, *P* = 0.449 for session interval; *n* = 73, *P* = 0.601 for stimulus pulses; two-tailed unpaired *t*-test; Table 1) between the groups.

**Figure 7:**
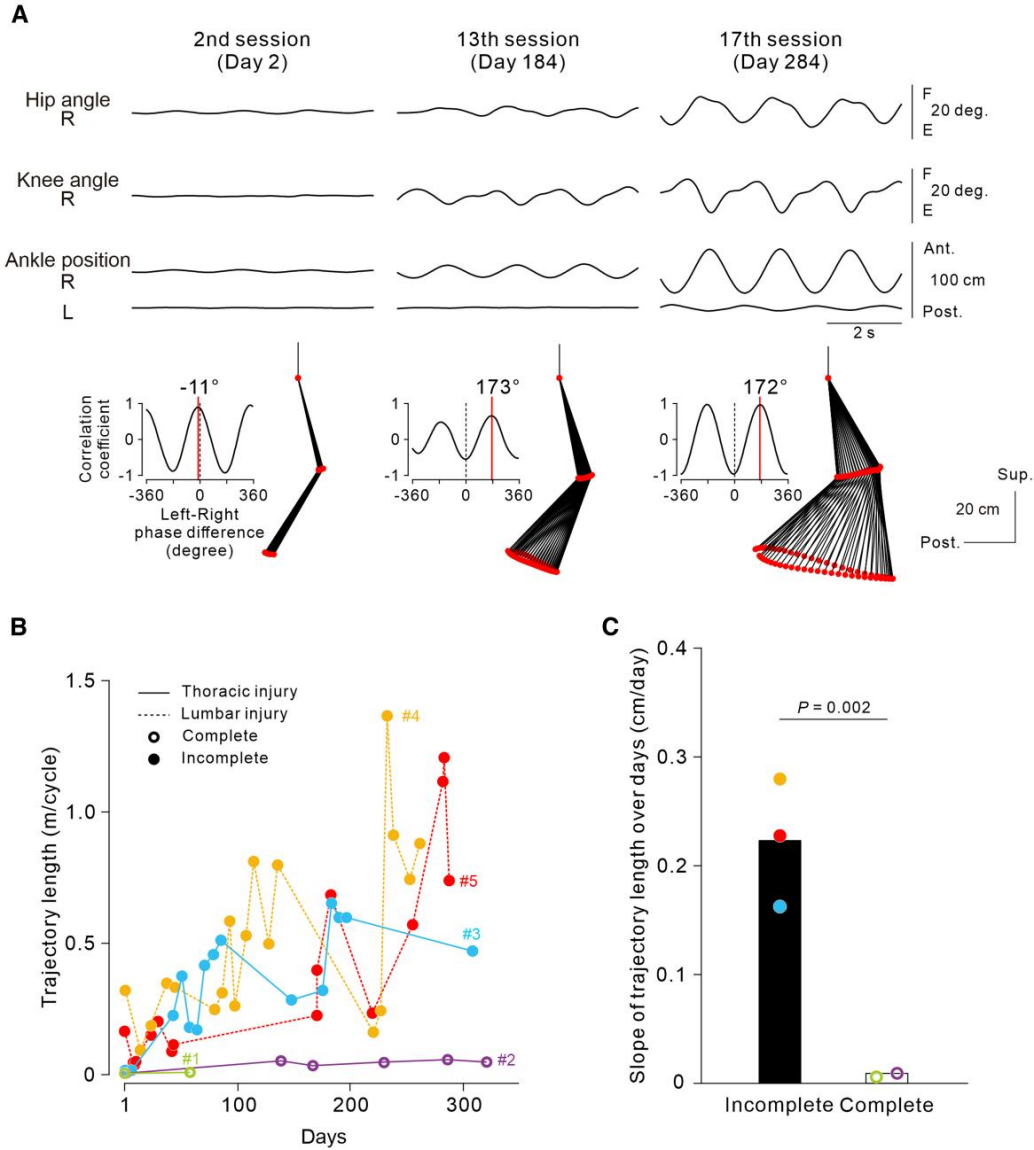
**Functional recovery of stimulation-free natural stepping performance.** (**A**) Representative examples of long-term improvement in natural voluntary stepping without artificial neural connection (ANC) (‘Vol.’) in Participant #5 with incomplete spinal cord injury (SCI) at the L1 level. The data were obtained from the first trial in each session (day). The arrangements of each trace are the same as those in Fig. 5A. (**B**) Recovery time course of trajectory length in all five participants. (**C**) Slope of trajectory length over days. The bars and circles represent the average and individual data, respectively, for the incomplete and complete SCI groups. The significant difference among groups was demonstrated by analysis of covariance performed on the dataset in ‘**B**’ with a generalized linear mixed-effects model (interaction between Day and Group, χ^2^ = 9.4609, *P* = 0.002162, *n* = 2, 6, 15, 16 and 19 for Participants #1, #2, #3, #4 and #5, respectively).

Figure 7A shows a representative example of the recovery time course of volitional cyclic stepping without the ANC (‘Vol.’) in Participant #5, who had incomplete loss of lower-limb function owing to lumbar SCI. Improvements in joint movement and trajectory length were observed over months (Fig. 7A and Supplementary Video 7). Improvements were also observed in Participants #3 (Supplementary Video 7) and #4, who had incomplete loss of lower-limb motor function after thoracic and lumbar SCI, respectively (Fig. 7B). Conversely, Participant #2, who had complete loss of lower-limb function after thoracic SCI, did not show robust improvement in natural stepping performance without the ANC interface (Fig. 7B). The difference in recovery between the incomplete and complete SCI groups was supported by the significant difference in the slope of the ankle trajectory length over time (χ^2^ = 9.4609, *P* = 0.0022; analysis of covariance with a generalized linear mixed-effects model; Fig. 7C). The same result was obtained in an analysis excluding Participant #1, who completed only a few ANC sessions (interaction between Day and Group, χ^2^ = 6.4636, *P* = 0.01101; analysis of covariance with a generalized linear mixed-effects model). From a practical point of view, we observed that Participant #3 was able to step forwards on the floor while standing with full body weight support from a bearing harness and long-limb orthoses after the 17th session of non-invasive ANC interface use (Day 312; Supplementary Video 8), although she was unable to make any stepping movements even in a gravity-free environment before the first session (Day 1 in Supplementary Video 7). She was still unable to move forwards by herself without external traction support. The manual muscle test indicated that leg extremity motor function was slightly improved in Participants #3, #4 and #5, who had incomplete loss of lower-limb motor function (Table 1).

In summary, these findings suggest that long-term non-invasive ANC application could be an effective bilateral leg training method for improving ANC-controlled cyclic stepping in participants with SCI whose lumbar locomotor circuits are spared from the lesion, in addition to volitional cyclic stepping without the ANC interface in participants with SCI whose descending motor pathways to the lumbar spinal cord are preserved.

## Discussion

The present findings provide proof of concept that the control of stepping movement in impaired legs can be regained using a non-invasive ANC interface for closed-loop magnetic stimulation of residual lumbar motor circuits in individuals with SCI. Furthermore, long-term application of this paradigm led to improvements in ANC-controlled cyclic stepping and induced functional recovery in natural voluntary stepping without any aid. These results indicate that the artificial descending motor commands created by non-invasive closed-loop magnetic spinal stimulation should compensate for interrupted descending pathway function and strengthen both the preserved spinal circuits and the descending pathways.

### Bypassing the interrupted descending pathways

Our ANC interface was designed to transfer voluntary commands to preserved lumbar spinal motor circuits while bypassing a spinal lesion. Muscle activity in an intact hand was converted to electrical pulses controlling the frequency of magnetic stimuli targeting the lumbar spinal motor circuits. In this context, fine voluntary commands could be delivered to the spinal motor circuits. Our recent work in monkeys with subcortical stroke showed that cortical neuronal activities in the facial motor area or the somatosensory area were able to manipulate paralysed hand muscle activity via an ANC interface,^22^ demonstrating that the ANC can provide cortical sites with new functions to govern anatomically unconnected spinal motoneurons and muscles. Hence, these findings justify the applicability of hand muscle-controlled magnetic spinal stimulation to transfer voluntary motor commands for lower-limb movements.

In line with our previous findings in able-bodied individuals,^18,19^ magnetic spinal stimulation targeting lumbar motor circuits induced bilateral alternate stepping even in participants with chronic SCI who could not perform any visible stepping voluntarily (Figs 1 and 4A). Although it is unclear whether magnetic spinal stimulation engages the lumbar spinal locomotor circuits, the findings suggest that the spinal lumbar circuits caudal to the lesion can generate asymmetric, bilateral leg stepping movements. We observed rhythmic activity patterns of leg muscles, which might underlie opposite directional left–right leg stepping, coincident with the phase of stimulus bursts. Although we lack sufficient evidence, we presume that biased summative activity across hip flexors and extensors in multiple hip joint muscles, such as the Iliop, Glut, adductor muscle group and tensor fascia femoris, in addition to the RF and BF muscles, might be involved in ANC-controlled alternate stepping movements, as we observed (Fig. 4G and H and Supplementary Fig. 2). The magnitude of leg stepping was often asymmetric, with greater movement observed on the side exhibiting hip flexion. This asymmetry raised the concern that the smaller hip extension movements observed on the contralateral side might be induced passively as counter-motions in response to the larger hip flexion movements. However, we excluded this possibility by showing that these hip extension movements were actively elicited by the stimulation, because they persisted even when the contralateral leg was mechanically constrained (Supplementary Fig. 3). These findings indicate that both hip flexion and extension were induced independently and actively by the stimulus, rather than being passive outcomes of interlimb mechanical interactions.

We demonstrated that all the participants succeeded in controlling cyclic stepping parameters, such as initiation, termination, magnitude and cadence, using the non-invasive ANC interface regardless of the level or completeness of the spinal injury (Fig. 2), indicating that as long as the lumbar spinal circuit is sufficiently preserved to induce left–right alternating stepping, even a small, non-invasive ANC interface could enable the participants to control stepping movements in their impaired legs. However, we also confirmed that ANC-controlled cyclic steps were obviously smaller in participants with lumbar lesions (Participants #4 and #5), in whom direct damage to the lumbosacral spinal cord impaired the function of lumbar motor circuits, including motoneurons, than in those with thoracic lesions (Participants #1, #2 and #3) (Figs 3D, 4D and 6C). These findings indicate that closed-loop magnetic spinal stimulation engaged the lumbar spinal circuits to produce bilateral alternate stepping. Therefore, for a greater effect from non-invasive ANC interface use, the lumbar spinal cord must be spared from direct damage as much as possible.

### Strengthening spinal circuits and cortical drive

Steps increased gradually over successive ANC-controlled cyclic stepping trials repeated on the same day (Fig. 3A and Supplementary Video 3) and continued to increase progressively when the participants repeated the ANC sessions over several months (Fig. 6 and Supplementary Video 6). Given that ANC-controlled stepping was performed while the lower limbs were fully relaxed, these improvements can hardly be explained by plastic changes at the cortical level and/or strengthened connectivity between the descending pathways and spinal circuits. The effects of repetition were clearly observed in participants with thoracic SCI, regardless of complete or incomplete loss of lower-limb function (Participants #2 and #3 in Fig. 3C; Participants #1, #2 and #3 in Fig. 6B), but not in participants with lumbar SCI (Participants #4 and #5 in Figs 3C and 6B). This difference between the two groups of participants can probably be explained by the fact that the lumbar motor circuits producing muscle activities for alternate stepping should be intact in participants with a thoracic lesion but might be involved, at least in part, in those with an L1 lesion. These findings suggest that the repetitive use of the non-invasive ANC interface over a few months induced plastic changes in the stimulated spared lumbar spinal circuits. The lumbar spinal cord is capable of adapting rapidly to variable environmental conditions even when descending inputs are disrupted.^23-25^ Thus, the retention of spinal circuit function in the lumbar spinal cord is crucial for improving the artificially constructed stepping ability via non-invasive ANC. It is also worth noting that improvements in muscle activity and stepping-like leg movement were observed predominantly in the leg exhibiting hip flexion in response to the stimulus bursts. Given that the trajectory length of stimulus-induced leg movement was larger in the hip flexion side, it is possible that the plastic effects induced by the repetition of non-invasive ANC trials were preferentially expressed in the spinal circuits supporting the hip flexor function.

Repeated use of the non-invasive ANC interface also induced functional recovery of stimulation-free natural stepping performance (‘Vol.’) (Figs 5 and 7). However, unlike ANC-controlled stepping, improvement in natural stepping was not observed clearly in the participant with complete loss of lower-limb function after thoracic SCI, in whom ANC-controlled stepping improved substantially (Participant #2 in Figs 6 and 7B). Conversely, robust improvement was observed in the participants with lumbar SCI, in whom ANC-controlled stepping did not improve clearly (Participants #4 and #5 in Figs 6B and 7B). These results suggest that different neural mechanisms from those underlying the improvement in ANC-controlled stepping must play a crucial primary role in the recovery of natural lower-limb motor function and exclude the contribution of mechanical factors such as increased muscle volume or reduced joint stiffness. A primary mechanism underlying the functional recovery of inherent lower-limb motor control might be plastic changes in the residual descending pathways to injury-spared lumbar cord neurons that can be attained by increased activity in supraspinal descending motor neurons and/or strengthened synaptic efficacy between descending motor axons and spinal neurons. Increased activity in cortical motor areas contributes to the recovery of motor function after SCI.^26,27^ Moreover, using an ANC, corticospinal transmission can be enhanced plastically by activity-dependent spinal stimulation, in which electrical spinal stimulation is delivered sustainably in association with the discharge of a corticospinal neuron for voluntary limb movement,^14^ indicating that an ANC can be a powerful tool for effectively inducing plastic neuromodulation at synapses between the descending pathways and spinal motor circuits. In the present study, multiple non-invasive ANC sessions were included in many ‘ANC + Vol.’ trials (Supplementary Table 1). Trajectory length in ANC-controlled stepping was most effectively facilitated in the leg exhibiting hip flexor activation when combined with voluntary effort (Fig. 4D). This was accompanied by selective enhancement of muscle activity in the corresponding agonist muscles, suggesting targeted modulation of spinal motor circuits (Fig. 4C). Although bilateral activation was observed occasionally, consistent alternation between the legs was not observed. These findings suggest that motor commands descending through spared pathways, although insufficient alone to evoke movement, can augment spinal excitability when paired with ANC-driven stimulation, thereby supporting partial restoration of stepping movement. Hence, it is likely that the spared lumbar motor circuits were co-activated repeatedly by both inputs from the spinal stimulation and residual descending commands.^28^ Over time, the transsynaptic efficacy of the descending pathways might have been enhanced in an activity-dependent manner accordingly, promoting functional improvement.

The extent of descending pathway preservation appears to be crucial for non-invasive ANC use to induce substantial recovery of natural motor function, which is supported by the present finding that stimulation-free natural stepping performance was not clearly improved in participants with thoracic SCI with complete loss of sensorimotor function (Participant #2 in Fig. 7B), although we detected a slight effect of descending commands on boosting ANC-controlled cyclic stepping (Fig. 4). In the incomplete SCI group (Participants #3, #4 and #5), we assumed that the descending pathways to the injury-spared lumbar cord motoneurons might be relatively spared, such that the increased descending drives resulting from strengthened synaptic efficacy in those pathways contributed to the recovery of natural lower-limb motor function. Unfortunately, we failed to detect visible muscle activity in the lower-limb muscles when the participants improved their stimulation-free natural stepping performance (see Results section). The inner muscles in the deep layer must play an important role in leg movement for locomotion.^29-32^ Given that we used surface wireless EMG sensors placed only over the superficially located RF and BF muscles (see Materials and Methods), key muscles primarily contributing to improved performance might not have been covered.

Although the improvement was observed bilaterally, the movement size was still asymmetric during stimulation-free natural voluntary stepping. We cannot rule out the possibility that the functional improvement achieved through our stimulation paradigm was limited, particularly in the hip extensor function. This asymmetry might be attributable to factors such as lateralized spinal damage and/or degeneration in neuromuscular tissues, which could have affected motor output between sides differentially.

For the participants with lumbar SCI, it is also important to clarify the extent to which the function of lumbar cord motoneurons and peripheral neuromuscular elements was preserved. We are aware that the lack of clinical neurophysiological assessments limits the interpretability of our results, and we will continue this research in another SCI population to investigate the neurophysiological mechanisms underlying the present findings.

According to the recovery curves (Figs 6B and 7B), substantial improvements were observed in stimulation-free natural voluntary stepping later than in ANC-controlled stepping. In addition, the detraining effects seemed to occur more frequently and to a greater extent in natural voluntary stepping, although performance did not return to initial levels during the intermittent sessions. We suppose that the extent of neural element preservation accounts for the abovementioned differences in recovery curves between ANC-controlled stepping and natural stepping. In Participant #3, who had thoracic lesions and showed substantial improvements in both ANC-controlled stepping and stimulation-free natural stepping, although the spinal neurons in the lumbar circuits might have been largely exempt from direct spinal damage, the nerve fibres in the descending pathways were certainly affected to some extent, suggesting that plastic neuromodulation in the residual descending pathways might be less effectively retained. This result is consistent with our finding that natural motor function was improved minimally in participants with complete SCI. In contrast to our study, previous studies reported that individuals who were clinically diagnosed with complete motor SCI at the cervical level regained voluntary leg muscle activity after long motor training (for >1 year) with open-loop epidural electrical spinal cord stimulation at the lumbar level.^33,34^ Therefore, for individuals with complete lesions above the lumbar cord, it might be difficult or take years to promote the recovery of natural motor function via non-invasive ANC interface use.

### Advantages of the present technique

The most significant achievement of the present study is that a fully non-invasive technique with a biomimetic closed-loop interface successfully restored control of bilateral alternate stepping movements in individuals with chronic SCI. Compared with other invasive spinal stimulation techniques, magnetic spinal stimulation has a lower risk of adverse events,^35^ which is supported by our recent study of individuals with chronic SCI and able-bodied individuals.^36^ Epidural spinal stimulation techniques have been used increasingly for post-SCI gait disorders,^17,34,37-40^ and approaches for establishing a closed-loop interface using these techniques require invasive surgical implantation of recording and stimulation electrodes.^17,39^ Therefore, closed-loop controlled magnetic spinal stimulation could serve as a non-invasive spinal stimulation therapy for people who have contraindications to or reject invasive electrode implantation surgery.

Notably, our closed-loop system is intuitive. Instead of brain activity, our interface requires only a single surface EMG channel from a muscle with preserved voluntary function as the input signal, which resolves the issue of inadequate real-time decoding capacity in surface EEG-based^12,41^ or invasive-recording^10,13,42^ interfaces for continuous control of cyclic bilateral stepping. The linear algorithm used to translate the amplitude of surface EMG signals into the frequency of stimulus trigger pulses replicates the natural population dynamics in corticospinal neurons and efficiently induces activity-dependent neuroplasticity in the stimulated spinal circuit. This algorithm is not only highly intuitive but also does not even require complex calibration of a decoding algorithm. Indeed, the present work and our previous studies in animals^7,9,22^ and humans^18^ demonstrated that naïve subjects require only a few minutes to learn this linear relationship between movement intention and stimulation-induced body movements. These high degrees of usability and safety are advantageous for clinical application.

### Perspective on clinical application

In addition to the findings of our previous studies of able-bodied individuals,^18,19^ the present study further demonstrates that closed-loop controlled magnetic spinal stimulation can feasibly be used to establish an artificial descending pathway for neurological gait disorders with chronic SCI to compensate for and strengthen the impaired descending pathways. However, it is not certain that the present findings of leg stepping movements fundamentally represent natural gait function. For practical application of this non-invasive ANC technique for gait rehabilitation in individuals with SCI, we must clarify further that the benefits can also be helpful for the ability to perform upright overground locomotion. We have previously shown that the non-invasive ANC technique is available for the induction of upright overground locomotion in able-bodied individuals.^18^ However, it is still unknown whether magnetic stimulation is also applicable to non-ambulatory SCI individuals with weak leg muscles. In this study, although no participants recovered to a self-ambulatory status, the participant with an incomplete thoracic lesion (#3) recovered to the point that she could step forwards on the floor in a standing posture with full body weight support (Supplementary Video 8). Thus, we expect that this technique would be more effective for the recovery of overground gait function in those with ambulatory SCI gait disorders. In these contexts, our non-invasive ANC technique could be best suited for individuals with SCI in whom the spinal lesion is located above the lumbar spinal cord and whose descending pathways are largely preserved.

Another remaining goal is to establish an optimal training regimen to maximize the benefits of non-invasive ANC interface use. The present study demonstrates that the repetition of ANC sessions progressively improved voluntary cyclic stepping performance with or without ANC use within a day (with ANC, Fig. 3; without ANC, Fig. 5) and over months (with ANC, Fig. 6; without ANC, Fig. 7). However, given that we tested a small number of participants for each incomplete and complete SCI, caution might be needed when interpreting our data. To reach a generalized conclusion, many participants need to be investigated in a future clinical trial. In addition, systematic investigations are needed to determine the specific training parameters that will promote neuroplasticity optimally (e.g. number and duration of trials, frequency, overall period) and the duration for which effects on parameters obtained through the above training are sustained. An optimal training interval determined by these findings would be valuable for maximizing the training effect and minimizing the detraining effect observed in the present study (Figs 6B and 7B). The above issues will be addressed in a future clinical trial.

## Supplementary Material

awaf230_Supplementary_Data

## Data Availability

The data that support the findings of this study are available from the corresponding author upon reasonable request.
