# Supplementary material for: Non-invasive closed-loop spinal stimulation restores leg stepping control in humans with paraplegia
Source: Brain. 2025 Nov 26;149(1):274–89. doi: 10.1093/brain/awaf230 (PMC12782173; doi:10.1093/brain/awaf230)
Supplement: awaf230_Supplementary_Data [file awaf230_supplementary_data.zip › brain-2024-01342-File010.pdf]

Supplementary materials

Supplementary methods

Non-invasive ANC interface

The paradigm of the ANC interface has been described elsewhere.[1–4] Briefly, to achieve an ANC that sends voluntary commands to the lumbar motor circuits and bypasses a spinal cord lesion via a non-invasive approach, EMG signals from the first dorsal interosseous (FDI) muscle were converted to stimulus pulses (Fig. 1A). These pulses were used to trigger magnetic stimuli delivered over the lumbar vertebrae (Fig. 1B). This non-invasive ANC was established using a computer interface designed to encode the outline of full-wave rectified and moving-averaged (250 ms window) surface EMG activity from a muscle and to convert the encoded EMG activity (X [a.u.]) into rectangular electrical pulses. The frequency of these pulses was determined by the level of EMG activity from the FDI muscle. Using output channels, participants were able to voluntarily control the initiation, termination, and frequency of magnetic stimulation through the interface. If the input muscle activity (X [a.u.]) was above the stimulus threshold (X_{th} [a.u.]), magnetic stimulation was triggered by modulating its frequency (f [Hz]) via the following equation:

$$f = f_0 + \frac{f_g}{X_{th}} \cdot (X - X_{th}), (f_0 \leq f \leq f_{Max}) \qquad (1)$$

where f_0 is the frequency at X_{th} (Hz), f_g is the stimulus frequency gain, and f_{Max} is the maximum frequency (Hz).

Prior to each session, we measured the background noise level and the amplitude of the input EMG activity; then, X_{th} and f_g were arbitrarily set by the experimenter. X_{th} was set as the value at which muscle activity could be detected without contamination from background signal noise or stimulus artefacts. The stimulus frequency gain (f_g) was also set as the value at which f_{Max} was obtained at the peak amplitude of the input EMG activity. f_0 was set at 1 Hz. f_{Max} was set at either 20 Hz or 25 Hz depending on the

stimulus responses for leg movements. These stimulus parameters effectively induced smooth leg behaviour.[1,4]

Magnetic stimulation was delivered over the lumbar vertebrae using a magnetic stimulator (Magstim Rapid; Magstim Co. Ltd., Whitland, UK) with a figure-eight (double 70 mm diameter) or circular (90 mm diameter) coil. The centre of the figure-eight coil or the upper edge of the circular coil was placed at the target intervertebral region. Before starting each day's session with non-invasive ANC, we determined the optimal stimulus site for inducing gait-like cyclic leg stepping in each participant. If the optimal spot was not determined at one specific position, multiple sites were investigated in a session (Supplementary Table 1). The stimulus intensity was gradually increased so that bilateral cyclic leg movements could be induced coincidently with the rhythm of hand gripping even at the slightest magnitude (Supplementary Table 1). When the participants experienced any unpleasant sensations, we did not increase the intensity further.

Recordings

To record muscle activity, bipolar surface electrodes (Trigno Wireless EMG; Delsys, MA, USA) were placed on the bellies of the rectus femoris (RF), the biceps femoris (BF) bilaterally, and the FDI muscle. EMG signals were amplified and bandpass filtered at 20–450 Hz. All signals were converted to digital data using an A/D converter at a sampling rate of 5 kHz for later off-line analysis (CED 1401 interface with Spike2 software; CED, Cambridge, UK). The stimulus-triggered averaging technique was used to quantify the muscle activity induced by the magnetic spinal stimulation.[4,5]

We recorded lower limb movements using a digital video camera (C905; Logicool, Tokyo, Japan) operated by Spike2 software. The camera was placed perpendicular to the floor. The frame rate and image resolution of the camera were 30 Hz and 640 × 480 pixels, respectively. Movement trajectories of the legs were detected by reflective markers at each of the following points: the acromion (shoulder), the lateral epicondyle of the humerus (elbow), the greater trochanter (hip), the lateral epicondyle of the femur (knee), and the lateral malleolus (ankle) (Fig. 1E). The marker trajectories were digitized at 30 Hz and smoothed (moving average with a 0.1-s interval) with Dipp-Motion XD software (DITECT, Tokyo, Japan). In some trials, video data were not stored on the

computer, but the EMG signals and stimulus triggers were stored. In these trials, we analysed only stimulus and hand EMG data.

Analysed sessions in the longitudinal experiments

Control of step length using the non-invasive ANC interface

For the experiment that investigated the controllability of step length, we analysed the trials in the 6th session (Day 57) for participant #1, the 7th session (Day 87) for participant #2, the 18th and 19th sessions (Days 1464 and 1514) for participant #3, and the 5th session (Day 45) for participant #4. Participant #5 was not tested.

Immediate improvement in ANC-controlled cyclic stepping by repetition

For the experiment that investigated the repetition effect of ANC trials on ANC-controlled stepping, the trials included for analyses were in the 6th and 11th sessions (Days 43 and 287) for participant #2; the 13th to 16th sessions (Days 177, 184, 191, 198) for participant #3; the 3rd and 6th to 9th sessions (Days 24, 80, 87, 94, 99) for participant #4; and the 10th to 12th sessions (Days 44 and 171) for participant #5. Participant 1 was not tested.

Voluntary effort boosts ANC-controlled cyclic stepping

For the experiment that investigated the effect of voluntary leg movement effort on ANC-controlled cyclic stepping, the analysed trials for each participant were performed in the 6th session (Day 57, #1), the 12th session (Day 287, #2), the 1st session (Day 1, #3), the 4th session (Day 38, #4), and the 5th session (Day 9, #5).

Immediate improvement in natural stepping after non-invasive ANC use combined with voluntary effort

We conducted the experiment that investigated the immediate after-effect of the combination of non-invasive ANC and voluntary effort on stimulation-free voluntary stepping in the 10th session (Day 168) for participant #2; the 6th, 7th and 9th to 11th

sessions (Days 51, 58, 72, 79, 86) for participant #3; the 5th and 10th to 12th sessions (Days 45, 108, 115, 129) for participant #4; and the 13th session (Day 184) for participant #5. Participant 1 was not tested.

Statistical analysis

As the experiments were exploratory, only data from relevant trials were included in the statistical analyses. Other trials that fell out of the statistical analyses were either recorded with incomparable conditions, including inconsistent stimulus intensity, site, or coil. Some trials that were intervened by an irrelevant ANC trial were also excluded from the analyses of three successive trials. To quantify the ankle trajectory length and the phase difference between left and right leg movements in the antero-posterior axis during cyclic stepping, we analysed leg kinematics for three consecutive stationary cycles. The normality of the data distributions was tested using the Shapiro–Wilk test. When normality could not be assumed, appropriate nonparametric statistical tests were conducted. For comparisons between short- and long-step conditions, two-tailed paired *t* tests were used to analyse hand EMG duration, step length, trajectory length, and step cadence. The Wilcoxon signed rank test was performed to analyse the number of stimuli. One-way ANOVA was performed to determine significant differences in ankle trajectory length among three successive trials (1st, 2nd, 3rd) in both ANC-controlled cyclic stepping ("ANC") and volitional cyclic stepping without ANC ("Vol."). The significance of the *F* values was obtained after Greenhouse–Geisser correction, if needed. Post hoc multiple comparisons were conducted using the Bonferroni corrected *t* test. Two-tailed unpaired *t* tests and one-sample *t* tests were also conducted to determine the significance of differences between participants with thoracic and lumbar SCI and between incomplete and complete SCI, respectively. Either parametric or Friedman one-way repeated-measures ANOVA was conducted to determine significant differences in the temporal characteristics of spinal stimulation among three successive trials of ANC-controlled cyclic stepping. Another one-way ANOVA with repeated measures was performed to determine significant differences in ankle trajectory length among the "Vol.", "ANC", and "ANC + Vol." tasks, followed by the post hoc multiple comparisons were conducted using the Bonferroni corrected *t* test. A generalized linear mixed effect model was used to statistically compare the differences in the stimulus-

triggered averaging EMGs between conditions "ANC" and "ANC + Vol.". As dependent variables, the EMG differences between conditions were fitted by the independent fixed effects of MUSCLE (RF vs. BF) and LEG (leg with hip flexion vs. leg with hip extension), the interaction of MUSCLE and LEG, and the random effect of each participant's intercept. To determine the significant effect of long-term ANC application on stepping performance, covariance analysis was performed with a generalized linear mixed-effects model. As dependent variables, the ankle trajectory length during ANC-controlled stepping and stimulation-free natural voluntary stepping was fitted with the independent fixed effects of DAY and GROUP (thoracic vs. lumbar, incomplete vs. complete), the interaction between DAY and GROUP, and the random effect of each participant's intercept. Statistical significance was set at alpha < 0.05. All pooled values are reported as the means ± standard deviations followed by ranges in the text.

ANC trial with recording pelvic muscle EMG signals

In the longitudinal experiment involving five SCI participants, leg muscle activity was recorded only from the RF and BF muscles. To examine the contribution of other hip flexors or extensors to ANC-controlled bilateral leg stepping, we additionally recorded EMGs from the pelvic muscles, which may contribute to hip joint motion when a non-invasive ANC interface is used. We enrolled 5 participants with chronic SCI (#6-#10, Table 1; IRB approval No. 17-2).

EMG signals were recorded in the bilateral iliopsoas[6] (Iliop) and gluteus maximus (Glut) in addition to the RF and BF using wireless EMG sensors (Trigno Wireless EMG; Delsys, MA, USA). Bilateral leg kinematics were measured using eight infrared cameras in a three-dimensional motion capture system (Flex3; OptiTrack, Inc., Corvallis, OR, USA) at a sampling frequency of 100 Hz.[4] The leg positions were detected by reflective markers on the major trochanters, knee, the malleolus of both legs, and the one side of shoulder. For the non-invasive ANC, surface EMG signals were recorded from the FDI muscle in a hand for trigger pulses. Magnetic spinal stimulation was delivered to an intervertebral region from Th11 to L5 at the intensity of 50% or 55% of the maximal stimulator output (Supplementary Table 1). The participants were instructed to perform rhythmic hand gripping to control bilateral alternative leg stepping using a non-invasive ANC interface.

Leg kinematics and EMGs were analysed for stable five consecutive cycles exhibiting the largest step lengths. Cross-correlation analysis between left and right ankle displacements in anterior-posterior axis was conducted to estimate the phase difference between left and right cyclic stepping movements. The range of stimulus artefacts for each stimulus pulse on EMG traces were detected by the stimulus-triggered averaging technique, and the EMG signals on the range were flattened out to remove the stimulus artefacts. For the Glut muscles, we identified muscle activity from stimulus artefact by confirming the following: 1) The magnetic stimulation over the lower back induced a high-frequency response followed by a relatively low-frequency biphasic response, whereas the stimulation over the side induced only a high-frequency response that gradually diminished within 10 ms after stimulus onset. 2) The magnitude of the later low-frequency response was independent of the magnitude of the earlier high-frequency response. 3) Palpable gluteal muscle twitch was observed with the stimulation over the lower back, but not with the stimulation over the side. That is, the presence or absence of the low-frequency biphasic response corresponded to the presence or absence of the gluteal muscle twitch, respectively. Based on these rationales, as stimulus artefacts, the earlier high-frequency responses were removed from the EMG signals to extract pure gluteal muscle activity (see, Supplementary Fig. 1). To quantify the muscle activity induced by the magnetic spinal stimulation, we measured the integral values of the rectified artefact-removed EMG traces in the period of stimulus bursts at each muscle. And then, hip flexor-extensor ratios were calculated for each leg by the following equation.

$$Hip\ flexor - extensor\ ratio = \left(\frac{EMG_{Iliop} - EMG_{Glut}}{EMG_{Iliop} + EMG_{Glut}} + \frac{EMG_{RF} - EMG_{BF}}{EMG_{RF} + EMG_{BF}}\right) / 2$$

(2)

where EMG_{Iliop}, EMG_{Glut}, EMG_{RF}, and EMG_{BF} are the integrals of rectified EMGs in the Iliop, Glut, RF, and BF muscles, respectively. As the motor threshold to the magnetic spinal stimulation was different across pelvic and thigh muscles,[5] the ratios were calculated separately for pelvic and thigh muscles and then averaged. Positive and negative values indicate the predominant activity of hip flexors and extensors, respectively. Two-tailed paired *t* test was conducted to compare between legs according to the direction of hip joint motion while stimulus bursts. The number of trials with

positive and negative values were separately counted for each leg and their populations were compared across sides by Fisher's exact probability test.

Control experiment to prove each side of stimulus-induced leg stepping

In another short-term experiment conducted in participants with complete SCI (#6, #7; IRB approval No. 25-1), we examined whether each side of leg stepping can be induced when the contralateral leg motion was mechanically restricted to dismiss the possibility that leg stepping movement in one side was artificially induced as counter reaction motion to the contralateral leg stepping movement. At the beginning, we confirmed that the participants were able to perform bilateral alternative leg stepping using non-invasive ANC interface. After that, the participants tried to perform same leg stepping movement while either side of leg was hold manually by an experimenter, and repeated it twice to examine leg kinematics in the unrestricted leg at each side. Trials with conditions of no leg restriction, left leg restriction, and right leg restriction were sequentially conducted as a set. For participant #6, the upper edge of the magnetic stimulation coil was located at an intervertebral region of L2-3 or L3-L4 in a set of trials. For participant #7, the coil was located with reference to L1-L2 intervertebral region. Stimulus intensity was 50% of maximal stimulator output for both participants. The experimental setting and kinematic analysis were same as the experiment recording pelvic muscle EMG signals. To compare the ankle trajectory length across conditions with and without leg restriction at each leg, the dependent variables of ankle trajectory lengths were fitted with the independent fixed effect of CONDITION (no restriction, restriction) and LEG (leg with hip flexion, leg with hip extension), the interaction between CONDITION and LEG, and the random effect of individual's intercept.

Supplementary figures

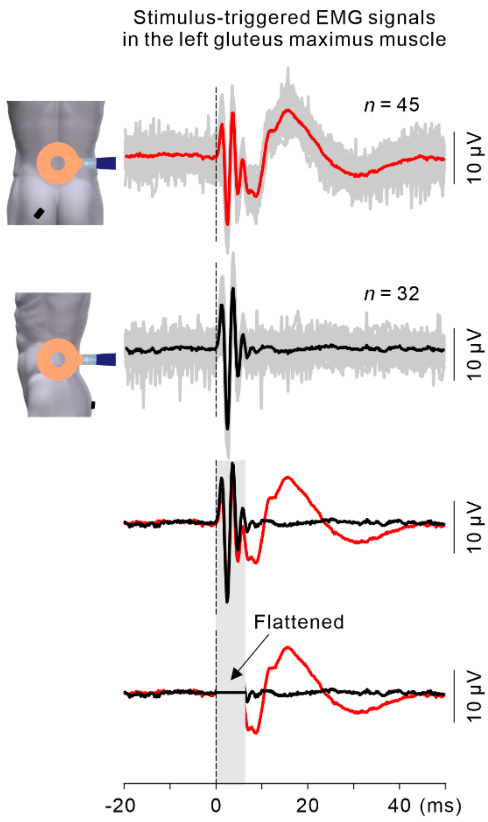

Supplementary Figure 1. Procedure to remove stimulus artifact from EMG signal in the gluteus muscle. Four panels show an example for the procedure to remove stimulus artefact from the EMG signal in the left gluteus maximus (Glut) muscle which was recorded in a prone posture on a bed. The traces on the 1st and 2nd rows show the EMG signals while the magnetic stimulation was delivered over the lower back and side, respectively at the intensity of 50% of maximum stimulator output. The stimulation over the lower back indued a palpable muscle twitch, but the stimulation over the side did not because of long distance between the stimulus coil and the lumbar spinal circuits. Superimposed average traces on the 3rd row show that the onset of biphasic muscle response was contaminated by preceding high-frequency stimulus artefact, but the artefact did not cover the entire muscle response. Therefore, only earlier high-frequency responses were flattened to extract pure muscle responses.

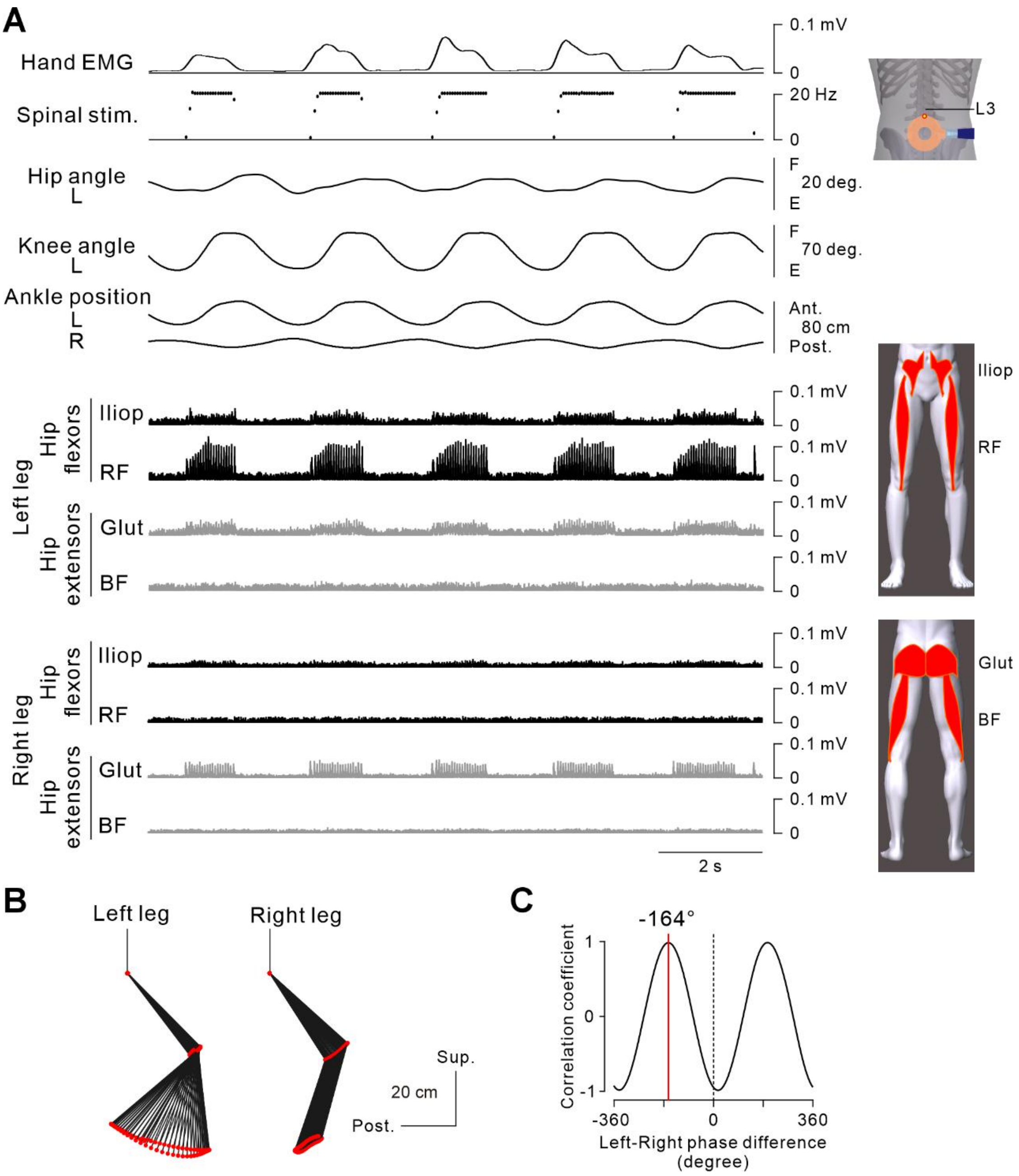

Supplementary Figure 2. Pelvic muscle activity during hand EMG-controlled cyclic leg stepping using the non-invasive artificial neural connection (ANC) interface. (**A**) Example of bilateral cyclic stepping using the non-invasive ANC interface in a participant with complete spinal cord injury (SCI) at the Th8 level (#6). Magnetic stimulation was delivered to the L3-L4 intervertebral region at an intensity of 50% of the maximum stimulator output. For this participant, we specifically recorded the EMGs from the bilateral pelvic muscles, which are particularly involved in hip flexion (Iliopsoas: Iliop) and extension (Gluteus maximus: Glue). Note that stimulus artefacts were removed from the EMG traces. (**B**) Stick pictures showing the left and

right leg kinematics at a stepping cycle in **A**. (**C**) Cross-correlogram of left and right leg movements at the anterior–posterior axis in the middle three successive cycles in **A**.

Result for Supplementary Figure 2

Although the participant #6 completely lost voluntary leg motor function bilaterally, she was able to perform bilateral alternative leg stepping using the non-invasive ANC interface (Supplementary Fig. 1A-C). The left and right paralyzed legs stepped forwards (hip flexion) and backwards (hip extension), respectively, while bursts of magnetic spinal stimulation were delivered. The magnitude of leg stepping was asymmetric with larger left leg stepping. The most pronounced activation in the left RF appeared to account for the left hip flexion at the stimulation phase. Although the activities in the thigh muscles (RF, BF) were not obvious in the right leg, we observed pronounced activity only in the right Glut muscle, which drove the hip extension motion.

In population-level data obtained in 5 participants with chronic SCI (#6-#10, Supplementary Table 2), we found bilateral alternative leg stepping in 33 trials (left-right phase difference, 150.9 ± 21.6 degrees). The magnitude of leg trajectory length was shown to be larger in the leg which moved forward while the bursts of magnetic spinal stimulation (71.15 ± 54.71 cm) than the other leg which moved backward (20.04 ± 13.95 cm; $n = 33$, $W = 523$, $P < 1.4713 \times 10^{-5}$, Wilcoxon signed rank test). However, there was no significant correlation in the leg trajectory length across sides ($n = 33$, $r = 0.1932$, $P = 0.2802$, Spearman's rank correlation analysis). Hip flexor-extensor ratios, estimated by muscle activity of bilateral Iliop, Glut, RF, BF muscles, showed significant asymmetry with corresponding to the hip joint motions (Fig. 1D). More than 80% of leg stepping directions induced by the magnetic spinal stimulation corresponded to the direction of hip joint motion that the dominant muscle activity served for (hip flexion leg, 84.85%; hip extension leg. 75.76%; Fig. 1E).

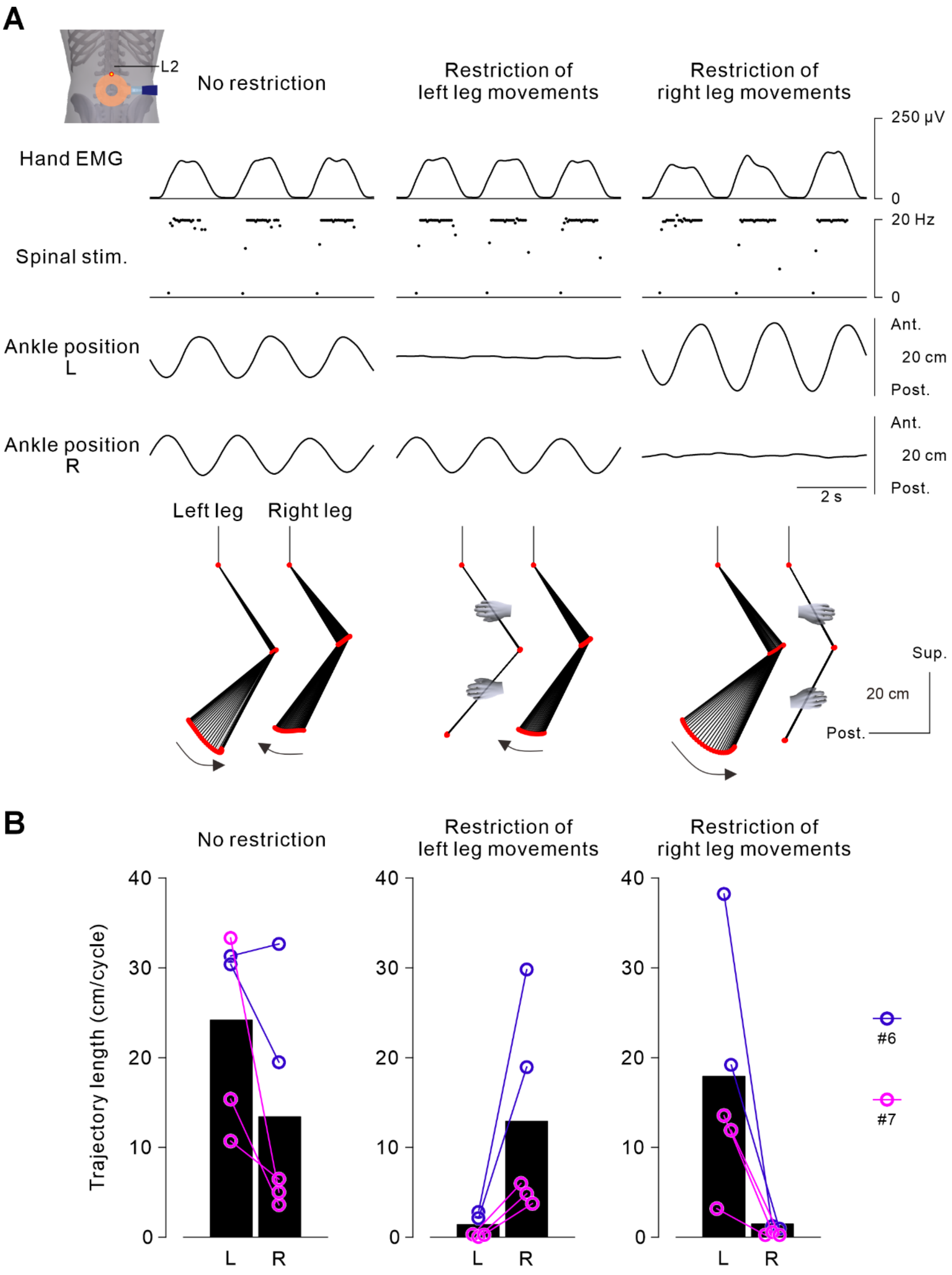

Supplementary Figure 3. Unilateral leg kinematics using non-invasive artificial neural connection (ANC) interface under the restriction of contralateral leg motion. (**A**) Examples of bilateral leg kinematics during ANC-controlled stepping in participant #6 with complete spinal cord injury (SCI) at the Th8 level. Magnetic stimulation was delivered to the L2-L3 intervertebral region at an intensity of 50% of the maximum

stimulator output. The trial was performed under no restriction of either leg movements (left), under the restriction of left leg movement (centre), and under the restriction of right leg movement (right). Stick pictures show the left and right leg kinematics while a stimulus burst. Arrows indicate the direction of leg stepping. (**B**) Ankle trajectory length at each leg in two participants with chronic SCI at the thoracic level (#6, deep blue; #7, magenta). Circles and black bars represent individuals' and mean data of all trials. In both participants, the left and right legs exhibited hip flexion and extension, respectively, while stimulus bursts.

Result for Supplementary Figure 3

In participant #6 with complete thoracic injury, the magnetic stimulation was delivered to L2-L3 intervertebral region at the intensity of 50% of maximal stimulator output. At the beginning, we confirmed that the participant was able to perform bilateral opposite directional leg stepping using non-invasive ANC interface (left-right phase difference, 171.0 degrees; Supplementary Fig. 2A left). In the identical noninavasive ANC setting, each of opposite directional leg stepping can be observed individually even when the contralateral side movement was mechanically restricted (Supplementary Fig. 2A, centre and right). In two participants with chronic SCI at the thoracic level (#6, #7), the stepping size was larger in the left leg exhibiting hip flexion during stimulus bursts than the right leg exhibiting hip extension (fixed effect of LEG, $X^2 = 11.4672$, $P = 0.0007084$). However, the stepping size were not statistically different across the conditions without and with mechanical leg restriction in either leg (fixed effect of CONDITION, X^2 = 0.0002, P = 0.9893639, interaction of CONDITION and LEG, X^2 = 1.7291, P = 0.1885269, generalize linear mixed effect model; Supplementary Fig. 3B).

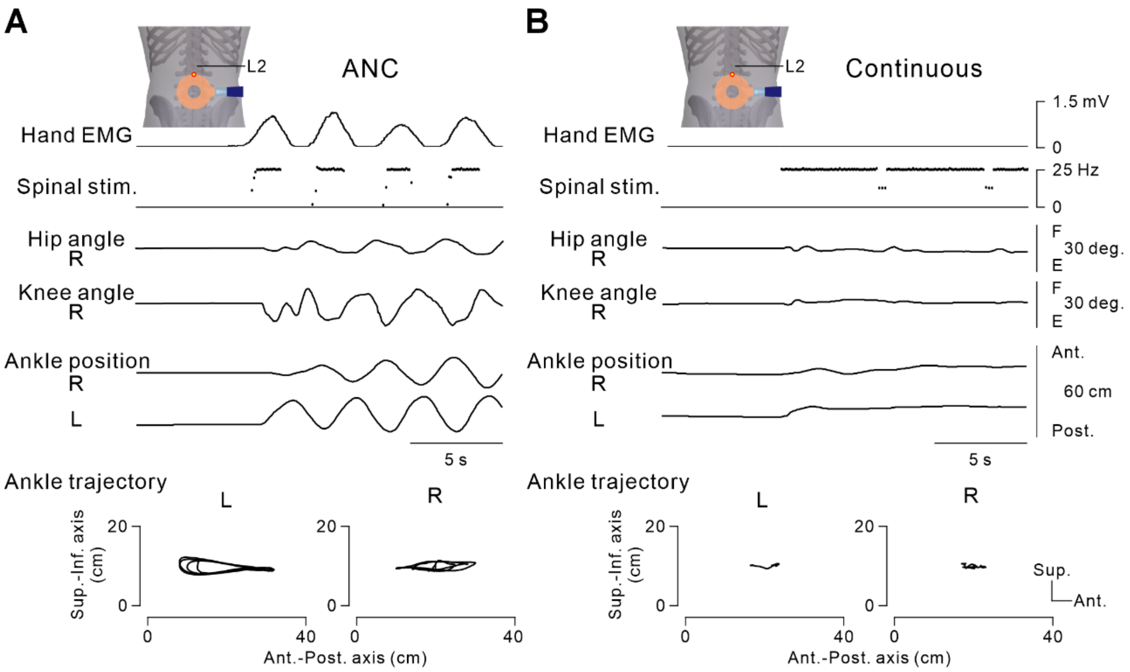

Supplementary Figure 4. Leg trajectory induced by two different patterns of magnetic spinal stimulation. Stimulation patterns, observed joint angles, and ankle trajectories: (**A**) stimulation controlled via the ANC interface using hand EMG signal and (**B**) continuous stimulation at a constant frequency. Both sets of data were obtained from participant #2 with complete spinal cord injury (SCI) at the Th3 level (4th session [Day 29]).

Result for Supplementary Figure 4

Magnetic stimulation was delivered to the L2-3 intervertebral space at 60% of the maximum stimulation intensity in both patterns of stimulation. Note that the magnetic pulses occasionally did not deliver continuously due to machine failure despite being triggered at a constant frequency of 25 Hz. Although the legs barely moved during the tonic stimulation phase, these unexpected stimulus frequency changes induced leg movements at the hip joint.

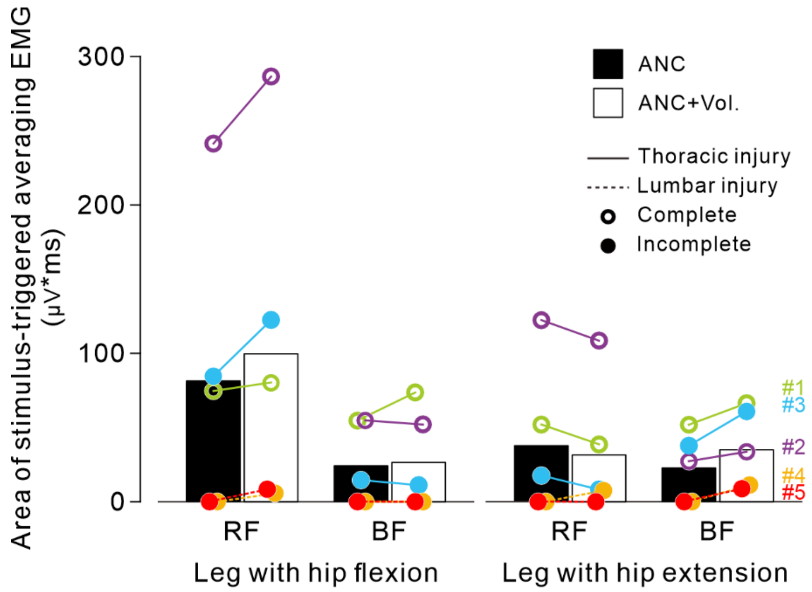

Supplementary Figure 5. Effect of voluntary effort on muscle activity during artificial neural connection (ANC)-controlled stepping. Area of stimulus-triggered averaging EMG in bilateral rectus femoris (RF) and biceps femoris (BF) muscles in the conditions of ANC-controlled stepping without ("ANC", black bar) and with voluntary effort ("ANC + Vol.", white bar). The circles represent individual participants.

Result for Supplementary Figure 5

For the trials for Figure 4 in the main text, we quantified the muscle activity by the area of stimulus-triggered averaging EMGs across participants. In the trial of stimulation-free natural voluntary stepping ("Vol."), none of the participants exhibited clear EMG activity in either RF or BF muscles bilaterally as shown in Figure 4B. In the participants with thoracic lesion (#1-#3), small but stimulus-evoked muscle responses were observed in bilateral RF and BF muscles in the trial of ANC alone ("ANC"). These responses became more pronounced under "ANC + Vol." condition, particularly in the agonist muscles – RF for the leg exhibiting hip flexion and BF for the leg with hip extension (Fig. 4C). In the participants with lumbar lesion (#4, #5), clear EMG responses were still not visible even during "ANC". However, the small responses emerged in a few muscles during "ANC + Vol." (Supplementary Fig. 5). Population-level analysis confirmed that additional voluntary effort enhanced muscle activation selectively in the agonistic muscles corresponding to the observed joint movement direction (i.e., RF for the leg with hip flexion, BF for the leg with hip extension; Fig. 4C).

Supplementary tables

Supplementary Table 1. Summary of testing sessions

			Number of trials[b]				Magnetic spinal stimulation[c]	
**Part.[a]**	**Session**	**Day**	**Vol.**	**ANC**	**ANC+Vol.**	**Total**	**Reference intervertebral level**	**Intensity**
#1	1	1	1	12	0	13	L1-L2, L2-L3, L3-L4	50, 55, 60
	2	8	0	15	0	15	L2-L3	65, 67
	3	36	0	7	0	7	L2-L3	60, 65, 70
	4	43	0	15	0	15	L2-L3, L3-L4, L4-L5	60, 65
	5	50	0	12	0	12	L2-L3, L3-L4, L4-L5	65
	6	57	2	9	1	12	L2-L3	60, 65
#2	1	1	1	11	0	12	L1-L2, L2-L3	55, 60
	2	8	0	4	0	4	Th12-L1, L1-L2	55
	3	22	0	12	0	12	Th12-L1, L2-L3, L3-L4, L4-L5	60, 65
	4	29	0	8	0	8	L2-L3, L3-L4	60, 70
	5	35	0	10	0	10	Th12-L1, L1-L2, L2-L3	65
	6	43	0	16	0	16	L1-L2, L2-L3, L3-L4	60, 65
	7	84	0	13	0	13	L3-L4, L4-L5	60
	8	112	0	15	5	20	Th12-L1, L1-L2, L2-L3, L3-L4, L4-L5	60
	9	140	1	0	19	20	L2-L3, L3-L4, L4-L5	60
	10	168	7	6	7	20	L3-L4, L4-L5	60
	11	231	2	16	2	20	Th12-L1, L1-L2, L2-L3, L3-L4	60
	12	287	2	1	2	5	L3-L4	60
	13	322	1	8	1	10	L3-L4	60
#3	1	1	2	6	10	18	L1-L2, L2-L3, L3-L4, L4-L5	40, 60, 70
	2	7	2	7	3	12	L3-L4	60, 70
	3	30	1	1	6	8	L3-L4	60
	4	37	1	18	2	21	Th12-L1, L1-L2, L2-L3, L3-L4, L4-L5	60
	5	44	22	4	5	31	L2-L2, L3-L4	60
	6	51	21	1	4	26	L3-L4	60
	7	58	27	5	3	35	L3-L4	60
	8	65	21	3	0	24	L3-L4	60
	9	72	18	3	3	24	L3-L4	60
	10	79	23	2	4	29	L3-L4	60
	11	86	28	1	5	34	L3-L4	60
	12	149	7	2	0	9	L2-L3	60
	13	177	18	4	0	22	L3-L4	60
	14	184	18	3	0	21	L3-L4	60
	15	191	17	3	0	20	L3-L4	60
	16	198	18	6	0	24	L3-L4	60
	17	310	19	3	2	24	L3-L4	60
	18	1464	0	15	0	15	Th12-L1	55
	19	1514	1	8	0	9	Th12-L1	55, 60
#4	1	1	1	16	3	20	Th12-L1, L1-L2, L2-L3, L3-L4, L4-L5	60
	2	15	4	8	6	18	Th12-L1, L1-L2	60
	3	24	14	4	0	18	Th12-L1	60
	4	38	5	1	6	12	Th12-L1	60
	5	45	17	3	4	24	Th12-L1	60
	6	80	17	3	4	24	Th12-L1	60
	7	87	16	3	3	22	Th12-L1	60
	8	94	18	5	4	27	Th12-L1	60
	9	99	20	3	5	28	Th12-L1	60
	10	108	18	1	7	26	Th12-L1	60
	11	115	18	2	6	26	Th12-L1	60

	12	129	19	2	3	24	Th12-L1	60
	13	136	3	3	6	12	Th12-L1	60
	14	221	15	5	4	24	Th12-L1	60
	15	228	19	0	6	25	Th12-L1	60
	16	234	19	4	5	28	Th12-L1	60
	17	239	16	5	5	26	Th12-L1	60
	18	254	16	4	3	23	Th12-L1	60
	19	263	16	2	7	25	Th12-L1	60
#5	1	1	0	16	0	16	Th12-L1, L1-L2, L2-L3, L3-L4, L4-L5	40, 45, 50, 60
	2	2	1	2	6	9	Th12-L1, L1-L2, L2-L3	55
	3	3	0	21	0	21	Th12-L1, L1-L2, L2-L3, L3-L4, L4-L5	55, 60
	4	8	2	5	2	9	Th12-L1, L1-L2	60
	5	9	1	12	3	16	Th12-L1, L1-L2, L2-L3, L3-L4	47, 50, 55
	6	10	7	1	8	16	Th12-L1, L1-L2, L2-L3	55, 60
	7	23	4	7	4	15	Th12-L1, L1-L2, L2-L3	55, 60
	8	30	5	0	7	12	Th12-L1	60
	9	43	16	4	6	26	Th12-L1	60
	10	44	11	5	1	17	Th12-L1	60
	11	171	16	4	0	20	Th12-L1	60
	12	172	21	5	4	30	Th12-L1	60
	13	184	5	3	3	11	Th12-L1	60
	14	221	17	1	4	22	Th12-L1	60
	15	256	17	4	0	21	Th12-L1	60
	16	283	16	8	0	24	Th12-L1, L2-L3	60
	17	284	14	2	6	22	Th12-L1	60
	18	289	15	2	5	22	Th12-L1	60
#6	1	1	0	10	0	10	Th12-L1, L1-L2, L2-L3, L3-L4, L4-L5	50
	2	2142	0	2	0	2	L2-L3, L3-L4	50
#7	1	1	0	10	0	10	Th11-Th12, Th12-L1, L1-L2, L2-L3, L3-L4, L4-L5	50
	2	2142	0	2	0	2	L1-L2	50
#8	1	1	0	4	0	4	Th11-Th12, Th12-L1, L1-L2,	55
#9	1	1	0	3	0	3	L1-L2	50
#10	1	1	0	6	0	6	Th12-L1, L1-L2, L2-L3, L3-L4, L4-L5	50

[a]Participants
[b]Trial numbers are counted in each trial type; trials with voluntary effort alone for leg stepping (Vol.), trials with non-invasive ANC alone (ANC), trials with non-invasive ANC combined with voluntary effort for leg stepping (ANC+Vol.) and in total. If multiple trial types were continuously tested in a single trial, each trial type was simultaneously counted.
[c]For magnetic spinal stimulation, the intervertebral level at which the reference point of the figure-eight coil (epicentre of wings) or circular coil (upper edge) was located and the intensity (% maximal stimulator output) are listed.

Supplementary Table 2. Magnetic spinal stimulation in three successive ANC-controlled cyclic stepping trials within one day

	1st trial	2nd trial	3rd trial
Training duration (s)	1.09 ± 0.33	1.04 ± 0.26	1.07 ± 0.25
Number of pulses/train	21.4 ± 6.3	20.5 ± 5.1	21.2 ± 5.0
Mean stimulus frequency (Hz/train)	18.5 ± 1.0	18.5 ± 0.8	18.9 ± 1.3
Intertrain interval (s)	0.99 ± 0.20	0.97 ± 0.14	0.95 ± 0.12

Characteristics of spinal stimulation were compared across three trials by Friedman one-way repeated-measures ANOVA (n = 14; train duration, X^2 = 3.000, P = 0.223; number of pulses per train, X^2 = 1.286, P = 0.526; mean stimulus frequency, X^2 = 0.143, P = 0.931) and parametric one-way repeated-measures ANOVA (intertrain interval, $F_{(2, 26)}$ = 0.366, P = 0.697).

Supplementary videos

Supplementary Video 1. Volitional control of gait-like cyclic bilateral leg stepping using the noninvasive ANC. A video of participant #2 recorded during the 4th session (Day 29). The data obtained from this video are shown in Figure 1D-G.

Supplementary Video 2. Volitional control of step length using the noninvasive ANC. A video of participant #1 recorded during the 6th session (Day 57). The data obtained from this video are shown in Figure 2A-C.

Supplementary Video 3. Improved ANC-controlled stepping by repeated ANC use within a day. A video of participant #3 recorded during the 8th session (Day 65). The data obtained from this video are shown in Figure 3A.

Supplementary Video 4. Effect of voluntary effort on ANC-controlled cyclic stepping. A video of participant #3 recorded during the 13th session (Day 177). The data obtained from this video are shown in Figure 4A.

Supplementary Video 5. Immediate improvement in stimulation-free natural voluntary stepping after ANC intervention with voluntary effort. First shown in the clip is a video of participant #4 recorded during the 11th session (Day 115). The data obtained from this video are shown in Figure 5A. Later shown in the clip is a video of participant #3 recorded during the 11th session (Day 86).

Supplementary Video 6. Improvement in ANC-controlled stepping over months. A video of participant #2 recorded during the 1st (Day 1), 4th (Day 29), and 10th sessions (Day 168). The data obtained from the video are shown in Figure 6A.

Supplementary Video 7. Functional recovery of stimulation-free natural stepping performance over months. First shown in the clip is a video of participant #5 recorded during the 2nd (Day 2), 13th (Day 184), and 17th (Day 284) sessions. The data obtained from this video are shown in Figure 7A. Later shown in the clip is a video of participant #3 recorded during the 1st (Day 1), 9th (Day 72), and 14th (Day 184) sessions.

Supplementary Video 8. Stimulation-free overground locomotion after long-term noninvasive ANC application. A video of participant #3 recorded on the day after the 17th session.

Supplementary references

1. Sasada S, Kato K, Kadowaki S, et al. Volitional walking via upper limb muscle-controlled stimulation of the lumbar locomotor center in man. *J Neurosci*. 2014;34(33):11131-11142.
2. Kato K, Sasada S, Nishimura Y. Flexible adaptation to an artificial recurrent connection from muscle to peripheral nerve in man. *J Neurophysiol*. 2016;115(2):978-991.
3. Kato K, Sawada M, Nishimura Y. Bypassing stroke-damaged neural pathways via a neural interface induces targeted cortical adaptation. *Nat Commun*. 2019;10(1):4699.
4. Kawai K, Tazoe T, Yanai T, Kanosue K, Nishimura Y. Activation of human spinal locomotor circuitry using transvertebral magnetic stimulation. *Front Hum Neurosci*. 2022;16:1016064.
5. Kawai K, Tazoe T, Yanai T, Kazuyuki K, Nishimura Y. Transsynaptic activation of human lumbar spinal motoneurons by transvertebral magnetic stimulation. *Neurosci Res*. Published online October 2, 2023. doi:10.1016/j.neures.2023.10.001
6. Jiroumaru T, Kurihara T, Isaka T. Establishment of a recording method for surface electromyography in the iliopsoas muscle. *J Electromyogr Kinesiol*. 2014;24(4):445-451.
